# Supplementary material for: The role of memory in non-genetic inheritance and its impact on cancer treatment resistance
Source: PLoS Comput Biol. 2021 Aug 30;17(8):e1009348. doi: 10.1371/journal.pcbi.1009348 (PMC8432806; doi:10.1371/journal.pcbi.1009348)
Supplement: S1 Text — Supporting mathematical analysis, tables, and figures. Fig A. Phenotypic switching probability and relative fitness gain. Figure A shows a representative form of the function βii(a) that represents the probability that a mother cell with phenotype i and age a bequeaths it’s phenotype to the daughter cells. Figure B shows the frequency dependent fitness increase function fn(θ) for n = 1, 2, 10 used to model fitness increases of the drug tolerant phenotype due to the Allee effect. Table A. The generic model parameters. The generic model parameters used to illustrate the impact of phenotypic switching on treatment resistance. Fig B. A comparison of growth rates for different growth functions fn, n = 1, 2, 3, 10, against Malthusian growth. The “no Allee” curves correspond to no frequency dependent fitness increase and fn = 1. Figure A shows the population evolution from an initial population comprised of 100 drug sensitive cells and one drug tolerant cell for the generic parameters in Table S1 obtained by simulating (S15). Figure B shows the population evolution from an initial population comprised of 1 drug tolerant cell and 100 drug tolerant cells for the generic parameters in Table A obtained by simulating (S15). Fig C. The proportion of drug-sensitive cells for increasing values of the sensitive cell death rate. A and B) the proportion of drug sensitive cells in the limited-resource setting is obtained by simulating the model (S2) for 500 days and computing of drug sensitive cells at day 500. A) the model predictions for PBBmax=0.05,0.13,0.22. B) the model predictions for σB = [1 × 10^−3^, 0.25, 0.5, 0.75] with PBBmax=0.6. Fig D. Fitting of the mathematical model to the Dingli et al. 2009 [60] data for a variety of fitting strategies. Figure A shows the fitting of the mathematical model (S2)to the Dingli et al. [60] data for the 8 different switching strategies given in the text. Figure B shows the regrouping of same 8 strategies after 2 applications of therapy. The p [file pcbi.1009348.s001.pdf]

Supporting Information: The role of memory in non-genetic inheritance and its impact on cancer treatment resistance

Tyler Cassidy[*,1] Daniel Nichol[2] Mark Robertson-Tessi[3] Morgan Craig[4,5] Alexander R.A. Anderson[*,3]

1 Theoretical Biology and Biophysics, Los Alamos National Laboratory, Los Alamos, NM 87545
2 Evolutionary Genomics and Modelling Lab, Centre for Evolution and Cancer, The Institute of Cancer Research, London, UK
3 Department of Integrated Mathematical Oncology, H. Lee Moffitt Cancer Center, Tampa, Florida, USA
4 Département de mathématiques et de statistique, Université de Montréal, Montreal, QC, Canada
5 CHU Sainte-Justine, Montreal, Canada
* tyler.cassidy@mail.mcgill.ca (TC); * Alexander.Anderson@moffitt.org (ARAA)

General model of phenotype switching

Here, we detail the mathematical model used in the main text. As mentioned, we consider two distinct cellular phenotypes A and B, where phenotype A represents the drug sensitive sub-population and phenotype B represents the drug tolerant sub-population. We denote the age density of cells with phenotype A at time t as $A(t,a)$, while $B(t,a)$ represents the density of cells of age a with phenotype B at time t. The object of clinical interest at time t is unlikely to be the density of cells with a given age, but rather total number of cells of each phenotype, given by

$$\bar{A}(t) = \int_0^\infty A(t,a)\mathrm{d}a \quad \text{and} \quad \bar{B}(t) = \int_0^\infty B(t,a)\mathrm{d}a. \tag{S1}$$

Under the assumptions in the main text, $A(t,a)$ and $B(t,a)$ satisfy the non-local PDE

$$\left.\begin{aligned} \partial_t A(t,a) + \partial_a A(t,a) &= -[d_A + R_A(\bar{A}(t),\bar{B}(t))]A(t,a) \\ \partial_t B(t,a) + \partial_a B(t,a) &= -[d_B + R_B(\bar{A}(t),\bar{B}(t))]B(t,a). \end{aligned}\right\} \tag{S2}$$

Cellular reproduction is incorporated through non-local boundary conditions. Specifically, reproduction of cells produces 2 daughter cells with age $a = 0$ that may not inherit the parent's phenotype. As mentioned, we assume that the probability of changing phenotypes depends on the age of the parent cell: i.e, older cells are more likely to switch phenotypes during reproduction [1,2]. We use $\beta_{ij}(a)$ to denote the probability that a cell with age a and phenotype i will create a cell of phenotype j during reproduction. Hence, the boundary condition corresponding to (S2) is

$$\left.\begin{aligned} A(t,0) &= 2\int_0^\infty R_A(\bar{A}(t),\bar{B}(t))\beta_{AA}(a)A(t,a) + R_B(\bar{A}(t),\bar{B}(t))\beta_{BA}(a)B(t,a)\mathrm{d}a \\ B(t,0) &= 2\int_0^\infty R_A(\bar{A}(t),\bar{B}(t))\beta_{AB}(a)A(t,a) + R_B(\bar{A}(t),\bar{B}(t))\beta_{BB}(a)B(t,a)\mathrm{d}a. \end{aligned}\right\} \tag{S3}$$

We model the probability that a cell of phenotype A gives birth to two cells of phenotype A as

$$\beta_{AA}(a) = P^*_{AA} + (P^{max}_{AA} - P^*_{AA})\exp\left[-\sigma_A a\right], \tag{S4}$$

while the probability of a cell of phenotype B producing two cells of phenotype B is

$$\beta_{BB}(a) = P^*_{BB} + (P^{max}_{BB} - P^*_{BB})\exp\left[-\sigma_B a\right]. \tag{S5}$$

We note that both $\beta_{AA}(a)$ and $\beta_{BB}(a)$ are non-negative decreasing functions of age. Moreover, as nascent cells are assumed to be restrained to either phenotype A or B, we necessarily have

$$\beta_{AB}(a) = 1 - \beta_{AA}(a) \quad \text{and} \quad \beta_{BA}(a) = 1 - \beta_{BB}(a).$$

We illustrate a representative form of $\beta_{ii}(a)$ in Fig A **A**. Using the bistable switch example, the σ_i parameters model the decay rate of the molecules that bias the switch. With $\sigma_i = 1 \times 10^{-2}$ days^{-1} (as is the case in our generic parametrization), a cell that replicates after 1 day will have a roughly 1% smaller probability of phenotypic inheritance than if the cell had replicated immediately upon birth. However, a precise value of σ_i is difficult to identify as there is a trade-off between the decay rate σ_i and the difference $P^{max}_{ii} - P^*_{ii}$. Therefore, we fix $\sigma_i = 10^{-2}$ throughout and included it as a parameter in our sensitivity analysis.

To complete the initial value problem defined by (S2), we prescribe initial conditions describing the age distribution of cells at time $t = 0$

$$A(0,a) = g_A(a) \quad \text{and} \quad B(0,a) = g_B(a).$$

It is natural to enforce that the initial age distributions $g_A(a)$ and $g_B(a)$ are non-negative functions. We note that cells with age $a > 0$ at time $t = 0$ must have been born at some time $s < 0$. However, $A(t, 0)$ and $B(t, 0)$ are only defined for $t > 0$. Nevertheless, it is possible to use the initial data of (S2) to define $A(-s, 0)$ and $B(-s, 0)$ for $s \in (0, \infty)$ by $A(-s, 0) = g_A(s) \exp([r_B + d_B]s)$ and $B(-s, 0) = g_B(s) \exp([r_B + d_B]s)$ [3], and we use these definitions when considering the equivalent renewal equation (S25).

Further, we are only interested in finite populations, so we require that $g_A, g_B \in L_1(0, \infty)$. The requirement that g_A and g_B are integrable ensures that (S2) has a unique solution in the sense of distributions, and that this solution belongs to $L_1(0, \infty)$ [4]. It follows that the quantities $\bar{A}(t)$ and $\bar{B}(t)$ are well-defined for $t > 0$. Rather than introducing a maximal age and subsequent mathematical complications in our simple model, we note that, along the characteristics of (S2) given by $a = t - t_0$,

$$\frac{\mathrm{d}}{\mathrm{dt}} A(t, a) = -[R_A(\bar{A}(t), \bar{B}(t)) + d_A] A(t, a), \quad \text{and}$$
$$\frac{\mathrm{d}}{\mathrm{dt}} B(t, a) = -[R_B(\bar{A}(t), \bar{B}(t)) + d_B] B(t, a).$$

both $A(t, a)$ and $B(t, a)$ decay exponentially in age. Nevertheless, given the biological interpretation of a in Eq. (S2), it may be reasonable to enforce a maximal cellular age a_{max}. However, translating this requirement to solutions of Eq. (S2) is not trivial.

Age structured PDE models similar to (S2) have been used extensively to model the progression of cells through a reproductive process [3–7], and there is extensive mathematical theory regarding the use of these age structured models in mathematical biology (see [8] for a review). As mentioned in the Main Text, other authors have considered PDEs structured in phenotype with non-local or diffusion terms. However, to our knowledge, the incorporation of the phenotypic switching in a McKendrick type equation is new.

Growth dynamics

To complete the mathematical model (S2), we now specify the form of R_A and R_B. In what follows, we explore multiple forms of these functions corresponding to different biological assumptions. We begin with the simplest case: unconstrained exponential growth which is appropriate in populations with (effectively) unlimited resources such as those that are continually replated during *in vitro* experiments. We then remove this assumption of unlimited resources and consider constrained growth, such as *in vitro* experiments that approach total confluence. Finally, we incorporate the effects of phenotypic cooperation, whereby a larger proportion of a certain phenotype can lead to increase phenotypic expansion through an Allee effect or frequency dependent fitness changes [9–13]. By considering the effects of different growth functions, we will explore the impact of growth stage on establishment of a drug tolerant population.

The simplest case corresponds to unconstrained growth, where there are unlimited resources in the environment. This surplus of resources allows for exponential, or Malthusian growth. In this case, we use a constant and phenotype dependent growth rate

$$R_A(\bar{A}(t), \bar{B}(t)) = r_A \quad \text{and} \quad R_B(\bar{A}(t), \bar{B}(t)) = r_B. \tag{S6}$$

During *in vitro* experiments, this unconstrained growth corresponds to the early growth phase of cells in culture or following the replating of an established cell culture into a nutrient rich environment.

In an environment with limited resources, the early exponential growth of a population gives way to tempered growth as competition for resources begins. This

restrained growth is typically modelled as logistic type growth. Thus, in the limited resource case, we model the growth rates as

$$\begin{aligned} R_A(\bar{A}(t),\bar{B}(t)) &= r_A\left(1-\frac{\bar{A}(t)+\bar{B}(t)}{K}\right) \quad \text{and} \\ R_B(\bar{A}(t),\bar{B}(t)) &= r_B\left(1-\frac{\bar{A}(t)+\bar{B}(t)}{K}\right). \end{aligned} \tag{S7}$$

We note that, as the population reaches the carrying capacity of the environment, the reproduction rates R_A and R_B both converge to 0.

Finally, we consider the influence of frequency dependent fitness increases in the B phenotype. This corresponds to cells of phenotype B gaining fitness as they become more populous and cooperate. This frequency dependent fitness increase, or Allee effect, has been observed in cancer [14–19]. To allow for cooperation amongst drug tolerant cells, we model reproduction as

$$\begin{aligned} R_A(\bar{A}(t),\bar{B}(t)) &= r_A\left(1-\frac{\bar{A}(t)+\bar{B}(t)}{K}\right) \quad \text{and} \\ R_B(\bar{A}(t),\bar{B}(t)) &= r_B\left(1-\frac{\bar{A}(t)+\bar{B}(t)}{K}\right) f_n(\bar{A}(t),\bar{B}(t)). \end{aligned} \tag{S8}$$

The function $f_n(\bar{A}(t),\bar{B}(t))$ models the increase in relative fitness of drug tolerant cells and determining a precise formulation of $f_n(\bar{A}(t),\bar{B}(t))$ is difficult [14]. However, from biological considerations, as the proportion of drug tolerant cells increases, the relative fitness of these cells should increase. Therefore, $f_n(\bar{A}(t),\bar{B}(t))$ should be monotonically decreasing in $\bar{A}$ and increasing in $\bar{B}$. In what follows, we will use the following frequency dependent growth function

$$\begin{aligned} f_n(\bar{A}(t),\bar{B}(t)) &= 1+\left(\frac{r_A-r_B}{r_B}\right)\left(\frac{\bar{B}(t)^n}{\bar{A}(t)^n+\bar{B}(t)^n}\right) \\ &= 1+\left(\frac{r_A-r_B}{r_B}\right)\left(\frac{\theta(t)^n}{1+\theta(t)^n}\right) = f_n(\theta(t)) \quad \text{for} \quad \theta(t)=\bar{B}(t)/\bar{A}(t). \end{aligned} \tag{S9}$$

The function $f_n(\bar{A}(t),\bar{B}(t))$ is a Hill-type function that ensures that $r_B f_n(\bar{A}(t),\bar{B}(t)))$ smoothly interpolates between r_B and r_A as the proportion of phenotype B cells in the total population increases between 0 and 1. The parameter n controls the steepness the sigmoidal curve. As n increases, the smooth sigmoid function approaches a step function at $\theta(t)=1$. Throughout the rest of this work, we consider $n=1,2,10$ to illustrate the impact of different frequency dependent fitness functions on population dynamics. In Fig A**B**, we show $f_1(\bar{A}(t),\bar{B}(t)), f_2(\bar{A}(t),\bar{B}(t))$ and $f_{10}(\bar{A}(t),\bar{B}(t))$ as a function of $\theta(t)$. We conclude by noting that the growth rates given by Equations (S6), (S7) and (S8) are non-negative functions for non-negative input $\bar{A}(t)$ and $\bar{B}(t)$.

Generic model of chemotherapy

Treatment necessarily imposes selection pressure against susceptible cells [14, 16, 20–22]. This selection pressure can drastically change population level dynamics and lead to the development and competitive release of resistant populations. As mentioned, this resistance can be driven by phenotypic switching [23–25]. Therefore, we include the effects of cytotoxic treatment in the mathematical model (S2). We emphasize that we are only attempting to model the qualitative effects of cytotoxic treatment and recall that, when incorporating therapeutic effects, we have assumed that cells of phenotype A

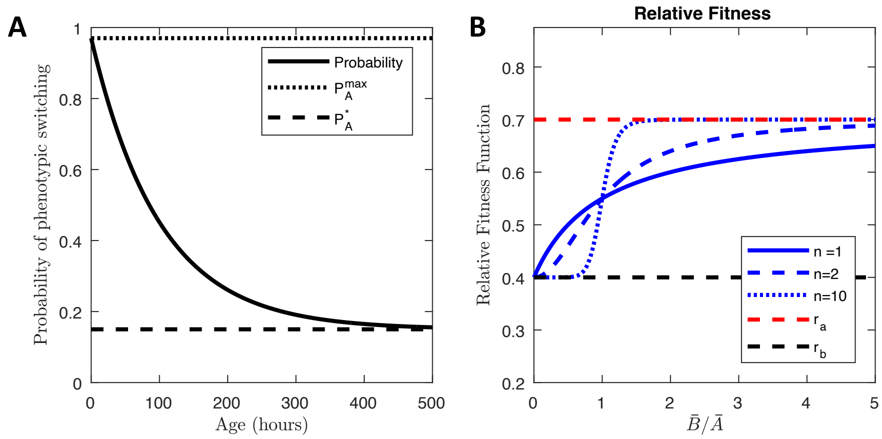

Fig A. Phenotypic switching probability and relative fitness gain. Fig **A** shows a representative form of the function $\beta_{ii}(a)$ that represents the probability that a mother cell with phenotype i and age a bequeaths it's phenotype to the daughter cells. Fig **B** shows the frequency dependent fitness increase function $f_n(\theta)$ for $n = 1, 2, 10$ used to model fitness increases of the drug tolerant phenotype due to the Allee effect.

are drug sensitive, while cells of phenotype B are drug tolerant and thus resistant to treatment.

We denote the concentration of a chemotherapeutic at time t as $C(t)$ and assume that therapy is given intravenously. The time dynamics of $C(t)$ are given by

$$\frac{\mathrm{d}}{\mathrm{dt}}C(t) = I(t) - k_{elim}C(t) \tag{S10}$$

where $I(t)$ is given by

$$I(t) = \begin{cases} \frac{Dose}{Vol \times T_{admin}} & \text{if} \quad t \in (t_i, t_i + T_{admin}) \\ 0 & \text{otherwise.} \end{cases}$$

and models the I.V administration of the cytotoxic drug during an injection time of T_{admin}, where Vol is the volume of distribution of the drug and $Dose$ is the size of one administration. The half life of the drug in question, $t_{1/2}$, defines the elimination constant through $k_{elim} = \log(2)/t_{1/2}$.

We assume that chemotherapy increases the death rate of drug sensitive cells through

$$d_A(t) = d_A + (d_A^{max} - d_A)\frac{C(t)}{C(t) + C_{1/2}},$$

where the half effect concentration is given by $C_{1/2}$. We note that, in our simple model, it is the ratio of the drug concentration and the half effect $C_{1/2}$ that completely determine the pharmacodynamics of the therapy in question. While using this simple pharmacodynamic model limits the direct applicability of our work, it allows for the identification of the crucial aspects in determining the effect of therapy.

Ordinary differential equations for $\bar{A}(t)$ and $\bar{B}(t)$

The partial differential equation (S2) is difficult to solve numerically. Moreover, we are primarily interested in the clinically relevant and biologically measurable $\bar{A}(t)$ and $\bar{B}(t)$. We note that the quantities of interest, $\bar{A}(t)$ and $\bar{B}(t)$, are independent of age.

Therefore, it is reasonable to expect that their dynamics should be determined by a system of two ODEs. As we will show, the age dependence in the non-local boundary condition (S3) will necessitate the inclusion of two extra ODEs. As the analysis that follows is identical for $\bar{B}(t)$, we only show the derivation of the ODE for $\bar{A}(t)$.

To derive the equivalent system of ODEs, we note that the formal solution of (S2) for $A(t, a)$ during treatment is

$$A(t,a) = A(t-a,0)\exp\left[-\int_{t-a}^{t} R_A(\bar{A}(s),\bar{B}(s)) + d_A + (d_A^{max} - d_A)\frac{C(s)}{C(s)+C_{1/2}}\mathrm{d}s\right], \tag{S11}$$

with a similar expression for $B(t, a)$. For notational convenience, we denote the total population of cells at time t, $\bar{A}(t) + \bar{B}(t)$ as $N(t)$.

We begin by using Leibniz's rule to differentiate (S1) and, after adding $0 = \partial_a A(t, a) - \partial_a A(t, a)$, find

$$\frac{\mathrm{d}}{\mathrm{dt}}\bar{A}(t) = -\left[R_A(\bar{A}(t),\bar{B}(t) + d_A + (d_A^{max} - d_A)\frac{C(t)}{C(t)+C_{1/2}}\right]\int_0^\infty A(t,a)\mathrm{d}a + A(t,0). \tag{S12}$$

The boundary conditions of (S2) give

$$\begin{aligned} A(t,0) &= 2R_A(\bar{A}(t),\bar{B}(t))\int_0^\infty \beta_{AA}(t,a)A(t,a)\mathrm{d}a \\ &\quad + 2R_B(\bar{A}(t),\bar{B}(t))\int_0^\infty \beta_{BA}(a)B(t,a)\mathrm{d}a \\ &= 2R_A(\bar{A}(t),\bar{B}(t))N_{AA}(t) + 2R_B(\bar{A}(t),\bar{B}(t))\int_0^\infty \beta_{BA}(a)B(t,a)\mathrm{d}a \end{aligned}$$

where

$$N_{AA}(t) = \int_0^\infty \beta_{AA}(a)A(t,a)\mathrm{d}a = \int_{-\infty}^{t} \beta_{AA}(t-a)A(t,t-a)\mathrm{d}a$$

with a similar expression for $N_{BB}(t)$. We note that, as $A(t, a) \in L_1(\mathbb{R}_+)$ with the normal Lebesgue measure, and $\beta_{AA} \leqslant 1$, it follows that N_{AA} is finite. Now, we recall that reproducing cells of phenotype i create either two cells of phenotype i or phenotype j. Therefore,

$$\beta_{AB}(a) = 1 - \beta_{AA}(a) \quad \text{and} \quad \beta_{BA}(a) = 1 - \beta_{BB}(a),$$

so

$$\begin{aligned} 2R_B(\bar{A}(t),\bar{B}(t))\int_0^\infty \beta_{BA}(a)B(t,a)\mathrm{d}a &= 2R_B(\bar{A}(t),\bar{B}(t))\left[\int_0^\infty (1-\beta_{BB}(a)B(t,a)\mathrm{d}a\right] \\ &= 2R_B(\bar{A}(t),\bar{B}(t))\left[\bar{B}(t) - N_{BB}(t)\right]. \end{aligned}$$

Thus, $\bar{A}(t)$ and $\bar{B}(t)$ satisfy a system of differential equations that require the evaluation of the integral terms $N_{AA}(t)$ and $N_{BB}(t)$, which is, once again, numerically challenging. Therefore, to implement (S12) numerically, we write $N_{AA}(t)$ and $N_{BB}(t)$ as the solutions of the differential equations

$$\begin{aligned} \frac{\mathrm{d}}{\mathrm{dt}}N_{AA}(t) &= \beta_{AA}(0)A(t,0) + \int_{-\infty}^{t}\frac{\mathrm{d}}{\mathrm{dt}}\left[\beta_{AA}(t-a)A(t,t-a)\right]\mathrm{d}a \\ &= \beta_{AA}(0)\left[2R_A(\bar{A}(t),\bar{B}(t))N_{AA}(t) + 2R_B(\bar{A}(t),\bar{B}(t))N_{BA}(t)\right] \\ &\quad + \int_{-\infty}^{t}[\frac{\mathrm{d}}{\mathrm{dt}}\beta_{AA}(t-a)]A(t,t-a)\mathrm{d}a - \left(R_A(\bar{A}(t),\bar{B}(t)) + d_I\right)N_{AA}(t) \end{aligned} \tag{S13}$$

an

$$\begin{aligned}\frac{\mathrm{d}}{\mathrm{dt}}N_{BB}(t) &= \beta_{BB}(0)B(t,0) + \int_{-\infty}^{t}\frac{\mathrm{d}}{\mathrm{dt}}\left[\beta_{BB}(t-a)B(t,t-a)\right]\mathrm{d}a\\ &= \beta_{BB}(0)\left[2R_A(\bar{A}(t),\bar{B}(t))N_{AB}(t) + 2R_B(\bar{A}(t),\bar{B}(t))N_{BB}(t)\right]\\ &\quad + \int_{-\infty}^{t}[\frac{\mathrm{d}}{\mathrm{dt}}\beta_{BB}(t-a)]A(t,t-a)\mathrm{d}a - \left(R_B(\bar{A}(t),\bar{B}(t)) + d_I\right)N_{BB}(t)\end{aligned} \tag{S14}$$

where we have used (S11) to write

$$A(t,t-a) = A(a,0)\exp\left[-\int_{a}^{t}R_A(\bar{A}(s),\bar{B}(s)) + d_A + (d_A^{max}-d_A)\frac{C(s)}{C(s)+C_{1/2}}\mathrm{d}s\right],$$

so that

$$\frac{\mathrm{d}}{\mathrm{dt}}A(t,t-a) = -\left(R_A(\bar{A}(t),\bar{B}(t)) + d_A + (d_A^{max}-d_A)\frac{C(t)}{C(t)+C_{1/2}}\right)A(t,t-a).$$

By using the following relationships for $\beta_{AA}(t-a)$ and $\beta_{BB}(t-a)$,

$$\begin{aligned}\frac{\mathrm{d}}{\mathrm{dt}}\beta_{AA}(t-a) &= \frac{\mathrm{d}}{\mathrm{dt}}\left(P_A^* + (P_A^{max}-P_A^*)\exp[-\sigma_A(t-a)]\right) = -\sigma_A\left(\beta_{AA}(t-a) - P_A^*\right)\\ \frac{\mathrm{d}}{\mathrm{dt}}\beta_{BB}(t-a) &= \frac{\mathrm{d}}{\mathrm{dt}}\left(P_B^* + (P_B^{max}-P_B^*)\exp[-\sigma_B(t-a)]\right) = -\sigma_B\left(\beta_{BB}(t-a) - P_B^*\right)\end{aligned}$$

we simplify (S13) to

$$\begin{aligned}\frac{\mathrm{d}}{\mathrm{dt}}N_{AA}(t) &= P_{AA}^{max}\left[2R_A(\bar{A}(t),\bar{B}(t))N_{AA}(t) + 2R_B(\bar{A}(t),\bar{B}(t))\left(\bar{B}(t) - N_{BB}(t)\right)\right]\\ &\quad - \left(R_A(\bar{A}(t),\bar{B}(t)) + d_A + (d_A^{max}-d_A)\frac{C(t)}{C(t)+C_{1/2}}\right)N_{AA}(t)\\ &\quad + \sigma_A\left(P_A^*\bar{A}(t) - N_{AA}(t)\right)\end{aligned}$$

while (S14) becomes

$$\begin{aligned}\frac{\mathrm{d}}{\mathrm{dt}}N_{BB}(t) &= P_{BB}^{max}\left[2R_A(\bar{A}(t),\bar{B}(t))\left(\bar{A}(t) - N_{AA}(t)\right) + 2R_B(\bar{A}(t),\bar{B}(t))N_{BB}(t)\right]\\ &\quad - \left(R_B(\bar{A}(t),\bar{B}(t)) + d_B\right)N_{BB}(t) - \sigma_B N_{BB}(t) + \sigma_B P_B^*\bar{B}(t).\end{aligned}$$

Thus, the system of ODEs for $\bar{A}(t)$ and $\bar{B}(t)$ is

$$\left.\begin{aligned}
\frac{\mathrm{d}}{\mathrm{dt}}\bar{A}(t) &= -[R_A(\bar{A}(t),\bar{B}(t)) + d_A + (d_A^{max} - d_A)\frac{C(t)}{C(t)+C_{1/2}}]\bar{A}(t) \\
&\quad + 2R_A(\bar{A}(t),\bar{B}(t))N_{AA}(t) + 2R_B(\bar{A}(t),\bar{B}(t))\left[\bar{B}(t) - N_{BB}(t)\right] \\
\frac{\mathrm{d}}{\mathrm{dt}}\bar{B}(t) &= -[R_B(\bar{A}(t),\bar{B}(t) + d_B]\bar{B}(t) + 2R_A(\bar{A}(t),\bar{B}(t))\left(\bar{A}(t) - N_{AA}(t)\right) \\
&\quad + 2R_B(\bar{A}(t),\bar{B}(t))N_{BB}(t) \\
\frac{\mathrm{d}}{\mathrm{dt}}N_{AA}(t) &= P_{AA}^{max}\left[2R_A(\bar{A}(t),\bar{B}(t))N_{AA}(t) + 2R_B(\bar{A}(t),\bar{B}(t))\left(\bar{B}(t) - N_{BB}(t)\right)\right] \\
&\quad - \left(R_A(\bar{A}(t),\bar{B}(t)) + d_A + (d_A^{max} - d_A)\frac{C(t)}{C(t)+C_{1/2}}\right)N_{AA}(t) \\
&\quad + \sigma_A\left(P_A^*\bar{A}(t) - N_{AA}(t)\right) \\
\frac{\mathrm{d}}{\mathrm{dt}}N_{BB}(t) &= P_{BB}^{max}\left[2R_A(\bar{A}(t),\bar{B}(t))\left(\bar{A}(t) - N_{AA}(t)\right) + 2R_B(\bar{A}(t),\bar{B}(t))N_{BB}(t)\right] \\
&\quad - \left(R_B(\bar{A}(t),\bar{B}(t)) + d_B\right)N_{BB}(t) - \sigma_B N_{BB}(t) + \sigma_B P_B^*\bar{B}(t).
\end{aligned}\right\} \tag{S15}$$

Initial conditions of the ODE model

The ODE (S15) is intrinsically finite dimensional with real valued initial conditions given by $\bar{A}(0)$, $\bar{B}(0)$, $N_{AA}(0)$ and $N_{BB}(0)$. This contrasts with the infinite dimensional system of PDEs (S2) with initial data given by $g_A(a)$ and $g_B(a)$ in the infinite dimensional space $L_1(0,\infty)$. To obtain the system of ODEs (S15), we partially solved the PDE (S2), so it is somewhat unsurprising that the resulting dynamical system is lower dimensional. We would like solutions of (S2) to correspond to solutions of (S15). Therefore, it is important to ensure that the initial conditions of the ODE system are appropriate. For integrable initial data $g_A(a)$ and $g_B(a)$ from the initial value problem (S2), it follows from the definition of $\bar{A}(t)$ and $\bar{B}(t)$ that

$$\bar{A}(0) = \int_0^\infty A(0,a)\mathrm{d}a = \int_0^\infty g_A(a)\mathrm{d}a \quad \text{and} \quad \bar{B}(0) = \int_0^\infty B(0,a)\mathrm{d}a = \int_0^\infty g_B(a)\mathrm{d}a.$$

We can easily see that the initial conditions of N_{AA} and N_{BB} must satisfy

$$N_{AA}(0) = \int_0^\infty \beta_{AA}(a)g_A(a)\mathrm{d}a \quad \text{and} \quad N_{BB}(0) = \int_0^\infty \beta_{BB}(a)g_B(a)\mathrm{d}a.$$

By the assumption that $g_A(a)$ and $g_B(a)$ are integrable and non-negative, these initial conditions are all finite and non-negative. In practice, it is simplest to assume that $g_A(a)$ and $g_B(a)$ are exponentially decaying functions of age so that the above integrals are simple to compute. This assumption of exponential decay in age is not unreasonable, both biologically and given the form of (S2). In the following section, we calculate the intrinsic growth rate of the mixed population, λ_p, by considering a population in exponential growth. As we will see, the assumption of exponential growth suggests that we set

$$g_A(a) = A_0\exp(-(r_A + d_A + \lambda_p)a) \quad \text{and} \quad g_B(a) = B_0\exp(-r_B + d_B + \lambda_p)a)$$

where we use different values of A_0 and B_0 to study the effects of initial population composition. We note that these definitions correspond to mapping the initial age

Parameter (unit)	Value
r_A (1/day)	0.7
r_B (1/day)	0.35
d_A (1/day)	0.1
d_A^{max} (1/day)	10
d_B (1/day)	0.1
P_{AA}^*	0.9
P_{AA}^{max}	0.95
K (cells)	10^4

Table A. The generic model parameters The generic model parameters used to illustrate the impact of phenotypic switching on treatment resistance.

distribution backwards along the characteristic curves [26]. From the definition of $g_A(a)$, we immediately obtain

$$\bar{A}(0) = \frac{A_0}{r_A + d_A + \lambda_p} \quad \text{and} \quad \bar{B}(0) = \frac{B_0}{r_B + d_B + \lambda_p}.$$

It therefore follows that

$$\begin{aligned} N_{AA}(0) &= \int_0^\infty \left(P_{AA}^* + (P_{AA}^{max} - P_{AA}^*)e^{-\sigma_A a}\right) A_0 \exp(-(r_A + d_A + \lambda_p)a)\mathrm{d}a \\ &= A_0 \left(\frac{P_{AA}^*}{r_A + d_A + \lambda_p} + \frac{P_{AA}^{max} - P_{AA}^*}{r_A + d_A + \lambda_p + \sigma_A} \right), \end{aligned}$$

and

$$N_{BB}(0) = B_0 \left(\frac{P_{BB}^*}{r_B + d_B + \lambda_p} + \frac{P_{BB}^{max} - P_{BB}^*}{r_B + d_B + \lambda_p + \sigma_B} \right).$$

Generic model parametrization

To study the role of phenotype switching on treatment resistance, we use a variety of physiologically based parameters rather than fitting the model to specific data. We assume that phenotype A cells successfully reproduce approximately once per day –similar to the reproductive time of most cells. Thus, we take $r_A = 0.7 \approx \log(2)/t_{A,2}$. Further, we assume that the phenotype B cells reproduce at about half the rate of phenotype A cells to account for the fitness cost of resistance [14, 27], and set $r_B = 0.35$. Unless otherwise stated, we fix $d_A = d_B = 0.01$. We note that, with these parameters, drug sensitive cells are fitter than drug tolerant cells. We set $P_{AA}^* = 0.9$ and $P_{AA}^{max} = 0.95$. While this parametrization is deliberately generic, we show later that our results are robust to parameter variation.

Model analysis

The mathematical model (S2) is quite simple and describes the time evolution of cell densities. In the following analysis, we do not consider the model with treatment, so $C(t) = 0$. We begin by demonstrating that solutions of (S2) evolving from non-negative and integrable initial data remain non-negative, as we would expect for a biological model.

Proposition A. *Let the model parameters be positive. Assume that $g_A(a)$ and $g_B(a)$ are integrable and almost-everywhere non-negative for $a \in (0, \infty)$. Then, the solution of* (S2) *is non-negative for all time $t > 0$ and all growth functions $R_i(\bar{A}(t), \bar{B}(t))$.*

Proof. Using the method of characteristics, the formal solution of (S2) is

$$\left.\begin{aligned} A(t,a) &= A(t-a,0)\exp\left[-\int_{t-a}^{t} d_A + R_A(\bar{A}(s),\bar{B}(s))\mathrm{d}s\right] \\ B(t,a) &= B(t-a,0)\exp\left[-\int_{t-a}^{t} d_B + R_B(\bar{A}(s),\bar{B}(s))\mathrm{d}s\right]. \end{aligned}\right\} \tag{S16}$$

We note that $A(t,a)$ and $B(t,a)$ preserve the sign of $A(t-a,0)$ and $B(t-a,0)$ respectively, and we recall that $A(t-a,0) = g_A(a-t)\exp(-[r_B+d_B](t-a))$ and $B(t-a,0) = g_B(a-t)\exp(-[r_B+d_B](t-a))$ for $t < a$. Thus, to show that $A(t,a) \geqslant 0$ and $B(t,a) \geqslant 0$, it is sufficient to show that $A(x,0) \geqslant 0$ and $B(x,0) \geqslant 0$ for all $x > 0$.

We consider $A(x,0)$, as the same argument holds for $B(x,0)$, and proceed by contradiction. Assume for contradiction that x^* is the first time such that $A(x^*,0) < 0$ or $B(x^*,0) < 0$, so $A(s,0) \geqslant 0$ and $B(s,0) \geqslant 0$ for all $s < x^*$. Now, we must have

$$\begin{aligned} \bar{A}(x^*) &= \int_0^\infty A(x^*,a)\mathrm{d}a \\ &= \int_0^\infty A(x^*-a,0)\exp\left[-\int_{x^*-a}^{x^*} R_A(\bar{A}(s),\bar{B}(s)) + d_A\mathrm{d}s\right]\mathrm{d}a \geqslant 0, \end{aligned}$$

and

$$\begin{aligned} \bar{B}(x^*) &= \int_0^\infty B(x^*,a)\mathrm{d}a \\ &= \int_0^\infty B(x^*-a,0)\exp\left[-\int_{x^*-a}^{x^*} R_B(\bar{A}(s),\bar{B}(s)) + d_B\mathrm{d}s\right]\mathrm{d}a \geqslant 0. \end{aligned}$$

Therefore, the functions $R_i(\bar{A}(x^*),\bar{B}(x^*))$ are non-negative for $i = A, B$. Furthermore, $\beta_{ij}(a) \geqslant 0$ from definition. Then, using the definition of $A(x,0)$ given in (S3) and the formal solution of (S2), we calculate

$$\begin{aligned} A(x^*,0) &= 2\int_0^\infty R_A(\bar{A}(t),\bar{B}(t))\beta_{AA}(a)A(t,a) + R_B(\bar{A}(t),\bar{B}(t))\beta_{BA}(a)B(t,a)\mathrm{d}a \\ &= 2\int_0^\infty R_A(\bar{A}(x^*),\bar{B}(x^*))\beta_{AA}(a)A(x^*-a,0)\exp\left[-\int_{x^*-a}^{x^*} R_A(\bar{A}(s),\bar{B}(s))d_A\mathrm{d}s\right]\mathrm{d}a \\ &\quad + \int_0^\infty R_B(\bar{A}(x^*),\bar{B}(x^*))\beta_{BA}(a)B(x^*-a,0)\exp\left[-\int_{x^*-a}^{x^*} R_B(\bar{A}(s),\bar{B}(s)) + d_B\mathrm{d}s\right]\mathrm{d}a \end{aligned}$$

Finally, since $x^* - a < x^*$, it follows that $A(x^*-a,0) \geqslant 0$ and $B(x^*-a,0) \geqslant 0$, so the integrals

$$\int_0^\infty 2R_A(\bar{A}(x^*),\bar{B}(x^*))\beta_{AA}(a)A(x^*-a,0)\exp\left[-\int_{x^*-a}^{x^*} R_A(\bar{A}(s),\bar{B}(s))d_A\mathrm{d}s\right]\mathrm{d}a$$

and

$$\int_0^\infty 2R_B(\bar{A}(x^*),\bar{B}(x^*))\beta_{BA}(a)B(x^*-a,0)\exp\left[-\int_{x^*-a}^{x^*} R_B(\bar{A}(s),\bar{B}(s)) + d_B\mathrm{d}s\right]\mathrm{d}a$$

are integrals of non-negative functions over a set of positive measure. Thus, $A(x^*,0)$ is the sum of two non-negative integrals and must satisfy $A(x^*,0) \geqslant 0$, a contradiction. The same argument for $B(x^*,0)$ yields the claim.

□

Turning now to the ODE model for $\bar{A}(t)$ and $\bar{B}(t)$ and having prescribed appropriate initial data, the theory of ODEs ensures that the initial value problem (IVP) defined by (S15) has a unique solution. Further, it follows immediately from Proposition A that solutions of (S15) evolving from non-negative initial data remain non-negative.

Nonlinear eigenproblem for the Malthusian parameter

To analyse the long term behaviour of the cell population, we search for a stable age distribution in the population [4,8]. This stable age distribution is equivalent to finding the first eigenelements of (S2) where we are considering the linearised version of (S2). We note that the linearised version of (S2) corresponds to purely Malthusian growth corresponding to constant R_A and R_B and effectively unlimited resources. We assume a solution of the type

$$A(t,a) = w(a)e^{\lambda_P t}, \quad B(t,a) = z(a)e^{\lambda_P t}. \tag{S17}$$

where λ_P is the Malthusian parameter to be determined [4]. The Malthusian parameter is an important quantity in population dynamics [5,28,29], and is typically used as measure of population fitness [10,30]. Later, we will show that the expected sign relationship between the Malthusian parameter λ_P and $R_0 - 1$, where the basic reproduction number R_0 is another classical measure of population fitness, holds in our model. This result normally follows immediately in most structured population models. However, the inclusion of phenotypic switching in our model complicates this relationship.

The unknown functions $w(a)$ and $z(a)$ are the age distributions of A and B respectively. These functions define a system of ordinary differential equations (ODEs) from which we will obtain a nonlinear eigenvalue problem with solution λ_P. For general λ, inserting the ansatz (S17) into (S2) yields the following system of ODEs

$$\left.\begin{aligned} e^{\lambda t}\left[w'(a) + \lambda w(a)\right] &= -[d_A + r_A]e^{\lambda t}w(a) \\ e^{\lambda t}\left[z'(a) + \lambda z(a)\right] &= -[d_B + r_B]e^{\lambda t}z(a), \end{aligned}\right\} \tag{S18}$$

with solutions

$$w(a) = A_0 \exp\left[-[d_A + r_A + \lambda]a\right], \quad z(a) = B_0 \exp\left[-[d_B + r_B + \lambda]a\right]. \tag{S19}$$

We must now ensure agreement with the boundary condition (S3) of the population PDE (S2), so

$$\left.\begin{aligned} e^{\lambda t}A_0 &= 2\int_0^\infty r_A\beta_{AA}(a)w(a)e^{\lambda t} + r_B\beta_{BA}(a)z(a)e^{\lambda t}\mathrm{d}a \\ e^{\lambda t}B_0 &= 2\int_0^\infty r_A\beta_{AB}(a)w(a)e^{\lambda t} + r_B\beta_{BB}(a)z(a)e^{\lambda t}\mathrm{d}a. \end{aligned}\right\}$$

Cancelling the $e^{\lambda t}$ terms gives a system of equations for the unknowns λ, A_0 and B_0

$$\left.\begin{aligned} A_0 = 2&\int_0^\infty r_A\beta_{AA}(a)A_0 \exp\left(-[d_A + r_A + \lambda]a)\right]\mathrm{d}a \\ &+ 2\int_0^\infty r_B\beta_{BA}(a)B_0 \exp\left(-[d_B + r_B + \lambda]a\right)\mathrm{d}a \\ B_0 = 2&\int_0^\infty r_A\beta_{AB}(a)A_0 \exp\left(-[d_A + r_A + \lambda]a\right)\mathrm{d}a \\ &+ 2\int_0^\infty r_B\beta_{BB}(a)B_0 \exp\left(-[d_B + r_B + \lambda]a\right)\mathrm{d}a. \end{aligned}\right\}$$

This linear system for the unknowns A_0, B_0 is equivalent to

$$\begin{bmatrix} A_0 \\ B_0 \end{bmatrix} = \begin{bmatrix} M_{11}(\lambda) & M_{12}(\lambda) \\ M_{21}(\lambda) & M_{22}(\lambda) \end{bmatrix} \begin{bmatrix} A_0 \\ B_0 \end{bmatrix}, \tag{S20}$$

where

$$M_{11}(\lambda) = 2\int_0^\infty r_A \beta_{AA}(a) \exp\left(-[d_A + r_A + \lambda]a\right) \mathrm{d}a \quad \text{and}$$
$$M_{22}(\lambda) = 2\int_0^\infty r_B \beta_{BB}(a) \exp\left(-[d_B + r_B + \lambda]a\right) \mathrm{d}a.$$

We note that newly born cells must be of either phenotype A or B, so we have

$$M_{21}(\lambda) = \int_0^\infty (1 - \beta_{AA}(a))\, r_A \exp\left(-[d_A + r_A + \lambda]a\right) \mathrm{d}a = \frac{2r_A}{d_A + r_A + \lambda} - M_{11}(\lambda)$$

and

$$M_{12}(\lambda) = \int_0^\infty (1 - \beta_{BB}(a))\, r_B \exp\left(-[d_A + r_A + \lambda]a\right) \mathrm{d}a = \frac{2r_B}{d_B + r_B + \lambda} - M_{22}(\lambda).$$

Consequently, the Malthusian parameter λ_P is the rightmost real solution of the nonlinear eigenproblem defined by (S20) and must satisfy

$$\det\left[M(\lambda) - I\right] = 0, \tag{S21}$$

where $M(\lambda)$ is given by the matrix in (S20). In the case of no phenotypic switching, the matrix $M(\lambda)$ is diagonal and this eigenvalue problem is simple. The following proposition is nearly obvious from the biological interpretation of the problem.

Proposition B. *Assume that cells cannot switch phenotype and that the model parameters are positive. Then, the Malthusian parameter is given by* $\lambda_P = \max[r_A - d_A, r_B - d_B]$.

Proof. If offspring directly inherit the phenotype of the parent cell, then $P_{ii}^{max} = P_{ii}^* = 1$. It follows that $M_{12} = M_{21} = 0$, and the matrix (S20) is given by

$$\begin{bmatrix} M_{11}(\lambda) & 0 \\ 0 & M_{22}(\lambda). \end{bmatrix}$$

The eigenvalues are therefore $M_{11}(\lambda)$ and $M_{22}(\lambda)$, which, for $P_i^{max} = P_i^* = 1$, are given by

$$M_{11}(\lambda) = \frac{2r_A}{\lambda + d_A + r_A} \quad \text{and} \quad M_{22}(\lambda) = \frac{2r_B}{\lambda + d_B + r_B}.$$

Then, 1 is an eigenvalue if and only if $\lambda = r_A - d_A$ or $\lambda = r_B - d_B$. The Malthusian parameter is the maximum of these values, so $\lambda_P = \max[r_A - d_A, r_B - d_B]$. □

It follows from the preceding proposition that, if no phenotypic switching can occur, a population comprised of entirely phenotype A cells has Malthusian parameter $\lambda_A = r_A - d_A$ and a population with only type B cells has Malthusian parameter $\lambda_B = r_B - d_B$. To simplify notation, we will assume, without loss of generality, that $\lambda_B \leqslant \lambda_A$.

We will now show that allowing for phenotypic switching acts to decrease population fitness. Namely, if λ_P is the Malthusian parameter of the switching

population, then $\lambda_P \in (\lambda_B, \lambda_A)$. We begin by removing the restriction that $P^*_{ii} = P^{max}_{ii} = 1$, and evaluate (S21). Then, this determinant becomes

$$0 = (1 - M_{11}(\lambda))(1 - M_{22}(\lambda)) - \left(\frac{2r_A}{d_A + r_A + \lambda} - M_{11}(\lambda)\right)\left(\frac{2r_B}{d_B + r_B + \lambda} - M_{22}(\lambda)\right). \tag{S22}$$

For β_{ii} given by (S4) and (S5), we calculate

$$\begin{aligned} M_{11,22}(\lambda) &= 2r_i \int_0^\infty \left[P^*_{ii} + (P^{max}_{ii} - P^*_{ii}) \exp(-\sigma_i a)\right] \exp\left[-(\lambda + d_i + r_i)a\right] \mathrm{d}a \\ &= \frac{2r_i P^*_{ii}}{\lambda + d_i + r_i} + \frac{2r_i(P^{max}_{ii} - P^*_{ii})}{\lambda + d_i + r_i + \sigma_i}. \end{aligned}$$

Then, equation (S22) becomes

$$\begin{aligned} 0 = 1 &- \frac{4r_A r_B}{(d_A + r_A + \lambda)(d_B + r_B + \lambda)} \\ &+ \left(\frac{2r_B}{r_B + d_B + \lambda} - 1\right)\left[\frac{2r_A P^*_{AA}}{\lambda + d_A + r_A} + \frac{2r_A(P^{max}_{AA} - P^*_{AA})}{\lambda + d_A + r_A + \sigma_A}\right] \\ &+ \left(\frac{2r_A}{r_A + d_A + \lambda} - 1\right)\left[\frac{2r_B P^*_{BB}}{\lambda + d_B + r_B} + \frac{2r_B(P^{max}_{BB} - P^*_{BB})}{\lambda + d_B + r_B + \sigma_B}\right] \end{aligned} \tag{S23}$$

To simplify notation in the following analysis, we set

$$\begin{aligned} F(\lambda) = 1 &- \frac{4r_A r_B}{(d_A + r_A + \lambda)(d_B + r_B + \lambda)} \\ &+ \left(\frac{2r_B}{r_B + d_B + \lambda} - 1\right)\left[\frac{2r_A P^*_{AA}}{\lambda + d_A + r_A} + \frac{2r_A(P^{max}_{AA} - P^*_{AA})}{\lambda + d_A + r_A + \sigma_A}\right] \\ &+ \left(\frac{2r_A}{r_A + d_A + \lambda} - 1\right)\left[\frac{2r_B P^*_{BB}}{\lambda + d_B + r_B} + \frac{2r_B(P^{max}_{BB} - P^*_{BB})}{\lambda + d_B + r_B + \sigma_B}\right], \end{aligned}$$

so that roots of $F(\lambda)$ correspond to solutions of (S23).

It remains to show that (S23) admits at least one real root. While $F(\lambda)$ may admit multiple real roots, the Malthusian parameter λ_P is the rightmost real root by convention. As we have seen, in models without phenotypic switching, it is typically fairly straightforward to prove that λ^* exists and is unique [4,8]. However, the phenotypic switching in (S2) results in the off diagonal terms of $M(\lambda)$ and complicates the analysis here, as the Malthusian parameter is no longer a strictly monotonic function of the parameters r_i, d_i. However, $F(\lambda)$ is eventually monotonic for $\lambda > 0$ large enough.

Lemma C. *$F(\lambda)$ is strictly increasing for $\lambda > \lambda_A = \max(\lambda_A, \lambda_B)$.*

Proof. To simplify notation in the proof, we write for $i = A, B$,

$$g_i(\lambda) = \frac{2r_i}{d_i + r_i + \lambda} \quad \text{and} \quad h_i(\lambda) = \frac{2r_i P^*_{ii}}{\lambda + d_i + r_i} + \frac{2r_i(P^{max}_{ii} - P^*_{ii})}{\lambda + d_i + r_i + \sigma_i},$$

so

$$F(\lambda) = 1 - g_A(\lambda) g_B(\lambda) + (g_A(\lambda) - 1) h_B(\lambda) + (g_B(\lambda) - 1) h_A(\lambda).$$

Then, differentiating $F(\lambda)$ and regrouping terms gives

$$\begin{aligned} F'(\lambda) &= g'_A(\lambda)\left(h_B(\lambda) - g_B(\lambda)\right) + g'_B(\lambda)\left(h_A(\lambda) - g_A(\lambda)\right) \\ &\quad + \left(g_A(\lambda) - 1\right) h'_B(\lambda) + \left(g_B(\lambda) - 1\right) h'_A(\lambda). \end{aligned}$$

It is clear that $g_i'(\lambda) < 0$ and $h_i'(\lambda) < 0$ for $\lambda > -\min(r_A + d_A, r_B + d_B)$. Further, for $\lambda > \max(r_A - d_A, r_B - d_B)$, we obtain $g_i(\lambda) - 1 < 0$ and

$$h_i(\lambda) \leqslant \frac{2r_i P_{ii}^{max}}{r_i + d_i + \lambda} \leqslant \frac{2r_i}{r_i + d_i + \lambda} = g_i(\lambda).$$

Therefore, each of the terms in $F'(\lambda)$ is the product of two non-positive functions, with

$$(g_A(\lambda) - 1)\, h_B'(\lambda) + (g_B(\lambda) - 1)\, h_A'(\lambda) > 0.$$

It follows that $F'(\lambda) > 0$ and $F(\lambda)$ is strictly increasing for $\lambda > \max(r_A - d_A, r_B - d_B)$. □

We continue by considering an extremely particular case, where both sub-populations have the same fitness. Consequently, phenotypic switching does not affect population fitness.

Lemma D. *Let the model parameters be positive. If $r_A - d_A = r_B - d_B$, then $\lambda_P = r_A - d_A = r_B - d_B$ is the Malthusian parameter.*

Proof. Evaluating (S22) at $\lambda^* = r_A - d_A = r_B - d_B$ gives

$$\begin{aligned}(1-M_{11}(\lambda^*))(1-M_{22}(\lambda^*)) - \left(\frac{2r_A}{d_A + r_A + \lambda^*} - M_{11}(\lambda^*)\right)\left(\frac{2r_B}{d_B + r_B + \lambda^*} - M_{22}(\lambda^*)\right) \\ = (1 - M_{11}(\lambda^*))(1 - M_{22}(\lambda^*)) - (1 - M_{11}(\lambda^*))\,(1 - M_{22}(\lambda^*)) = 0.\end{aligned}$$

Then, since $F(\lambda)$ is strictly increasing for $\lambda > r_A - d_A$, λ^* is the rightmost real root of F, and $\lambda_P = \lambda^* = r_A - d_A$. □

We now consider the more general case, where $r_A - d_A \neq r_B - d_B$.

Lemma E. *Let the model parameters be positive and assume that $\lambda_A > \lambda_B > -\min[r_A + d_A, r_B + d_B]$. Then, there exists a real root λ^* of* (S23) *with $\lambda^* \in (\lambda_B, \infty)$.*

Proof. We begin by noting $F(\lambda)$ is continuous and well-defined for $\lambda \in (-\min[r_A + d_A, r_B + d_B], \infty)$. Further,

$$\lim_{\lambda \to \infty} F(\lambda) = 1 > 0. \tag{S24}$$

Now, recall that $\lambda_B = r_B - d_B$, so $r_B + d_B + \lambda_B = 2r_B$ and calculate

$$\begin{aligned}F(\lambda_B) &= 1 - \frac{2r_A}{(d_A + r_A + \lambda_B)}\frac{2r_B}{(d_B + r_B + \lambda_B)} \\ &\quad + \left(\frac{2r_B}{r_B + d_B + \lambda_B} - 1\right)\left[\frac{2r_A P_{AA}^*}{\lambda_B + d_A + r_A} + \frac{2r_A(P_{AA}^{max} - P_{AA}^*)}{\lambda_B + d_A + r_A + \sigma_A}\right] \\ &\quad + \left(\frac{2r_A}{r_A + d_A + \lambda_B} - 1\right)\left[\frac{2r_B P_{BB}^*}{\lambda_B + d_B + r_B} + \frac{2r_B(P_{BB}^{max} - P_{BB}^*)}{\lambda_B + d_B + r_B + \sigma_B}\right] \\ &= 1 - \frac{2r_A}{d_A + r_A + \lambda_B} + \left(\frac{2r_A}{r_A + d_A + \lambda_B} - 1\right)\left[P_{BB}^* + \frac{2r_B(P_{BB}^{max} - P_{BB}^*)}{\lambda_B + d_B + r_B + \sigma_B}\right] \\ &= \left(1 - \frac{2r_A}{d_A + r_A + \lambda_B}\right)\left(1 - \left[P_{BB}^* + \frac{2r_B(P_{BB}^{max} - P_{BB}^*)}{\lambda_B + d_B + r_B + \sigma_B}\right]\right).\end{aligned}$$

Now, since $\sigma_B > 0$, $\lambda_B + d_B + r_B + \sigma_B = \sigma_B + 2r_B > 2r_B$ and

$$\left[P_{BB}^* + \frac{2r_B(P_{BB}^{max} - P_{BB}^*)}{\lambda_B + d_B + r_B + \sigma_B}\right] < P_{BB}^{max} \leqslant 1$$
$$\Rightarrow \left(1 - \left[P_{BB}^* + \frac{2r_B(P_{BB}^{max} - P_{BB}^*)}{\lambda_B + d_B + r_B + \sigma_B}\right]\right) > 0.$$

By the assumption that $\lambda_B < \lambda_A$, we have $r_A + d_A + \lambda_B < 2r_A$. It follows that

$$\left(1 - \frac{2r_A}{d_A + r_A + \lambda_B}\right) < 0.$$

Thus, $F(\lambda_B) < 0$ and the intermediate value theorem yields the claim. □

We now regroup Lemmas C and E to establish the existence of the Malthusian parameter.

Theorem F. *Let the model parameters be positive. Then the Malthusian parameter λ_P of the population with phenotypic switching satisfies $\lambda_P \in (\lambda_B, \lambda_A)$.*

Proof. The existence and lower bound follows immediately from Lemma C and Lemma E. It only remains to show that $\lambda_P < \lambda_A$. Recalling that $\lambda_A = r_A - d_A$, we calculate

$$\begin{aligned}
F(\lambda_A) &= 1 - \frac{2r_A}{(d_A + r_A + \lambda_A)} \frac{2r_B}{(d_B + r_B + \lambda_A)} \\
&\quad + \left(\frac{2r_B}{r_B + d_B + \lambda_A} - 1\right)\left[\frac{2r_A P_{AA}^*}{\lambda_A + d_A + r_A} + \frac{2r_A(P_{AA}^{max} - P_{AA}^*)}{\lambda_A + d_A + r_A + \sigma_A}\right] \\
&\quad + \left(\frac{2r_A}{r_A + d_A + \lambda_A} - 1\right)\left[\frac{2r_B P_{BB}^*}{\lambda_A + d_B + r_B} + \frac{2r_B(P_{BB}^{max} - P_{BB}^*)}{\lambda_A + d_B + r_B + \sigma_B}\right] \\
&= 1 - \frac{2r_B}{d_B + r_B + \lambda_A} + \left(\frac{2r_B}{r_B + d_B + \lambda_A} - 1\right)\left[P_{AA}^* + \frac{2r_A(P_{AA}^{max} - P_{AA}^*)}{\lambda_A + d_A + r_A + \sigma_A}\right] \\
&= \left(1 - \frac{2r_B}{d_B + r_B + \lambda_A}\right)\left(1 - \left[P_{AA}^* + \frac{2r_A(P_{AA}^{max} - P_{AA}^*)}{\lambda_A + d_A + r_A + \sigma_A}\right]\right).
\end{aligned}$$

From the definition of λ_A, it follows that $\lambda_A + d_A + r_A + \sigma_A > 2r_A$, so

$$\left(1 - \left[P_{AA}^* + \frac{2r_A(P_{AA}^{max} - P_{AA}^*)}{\lambda_A + d_A + r_A + \sigma_A}\right]\right) > (1 - P_{AA}^{max}) \geqslant 0.$$

Moreover, $\lambda_A + r_B + d_B = r_A - d_A + r_B + d_B > r_B - d_B + r_B + d_B = 2r_B$, so

$$\left(1 - \frac{2r_B}{d_B + r_B + \lambda_A}\right) > 0.$$

Thus, $F(\lambda_A)$ is the product of two positive terms which ensures that $F(\lambda_A) > 0$. The intermediate value theorem ensures that there is at least one root $\lambda_i^* \in (\lambda_B, \lambda_A)$. Finally, Lemma C ensures that $F(\lambda)$ has no real roots $\lambda > r_A - d_A$. Then, the Malthusian parameter is the maximum of the possible roots $\{\lambda_i^*\}$ in the interval (λ_B, λ_A), so $\lambda_P = \max\{\lambda_i^*\} \in (\lambda_B, \lambda_A)$.

□

The basic reproduction number

As previously mentioned, there typically is a correspondence between the Malthusian parameter λ_P and the basic reproduction number. The basic reproduction number is the spectral radius of the next generation operator [31], and is typically understood as the expected number of new cells produced by each existing cell. We now recast the nonlinear eigenproblem (S20) as a renewal type equation from which we derive the basic reproduction number. In what follows, we use the formal solution of (S2) given by (S16).

To simplify notation, define

$$\begin{aligned} f_{11}(a) &= r_A \beta_{AA}(a) \exp[-(r_A + d_A)a], \quad f_{12}(a) = r_B \beta_{BA}(a) \exp[-(r_B + d_B)a], \\ f_{21}(a) &= r_A \beta_{AB}(a) \exp[-(r_A + d_A)a], \quad \text{and} \quad f_{22}(a) = r_B \beta_{BB}(a) \exp[-(r_B + d_B)a], \end{aligned}$$

and note that the boundary terms $A(t,0)$ and $B(t,0)$ are functions of time. We recall that we extended the initial conditions $g_A(a)$ and $g_B(a)$ to define $A(t-a,0)$ and $B(t-a,0)$ for $t < a$ by $A(t-a,0) = g_A(a-t)\exp(-[r_B + d_B](t-a))$ and $B(t-a,0) = g_B(a-t)\exp(-[r_B + d_B](t-a))$.

Now, inserting the formal solution (S16) into the boundary condition (S3), we see that $A(t,0)$ and $B(t,0)$ satisfy the renewal equation

$$\left.\begin{array}{rcl} A(t,0) &=& (A(t,0) * f_{11})(t) + (B(t,0) * f_{12})(t) \\ B(t,0) &=& (A(t,0) * f_{21})(t) + (B(t,0) * f_{22})(t). \end{array}\right\} \tag{S25}$$

Taking the Laplace transform of (S25) gives the linear system

$$\begin{bmatrix} \hat{A}(t,0) \\ \hat{B}(t,0) \end{bmatrix} = \begin{bmatrix} \hat{f}_{11}(\lambda) & \hat{f}_{12}(\lambda) \\ \hat{f}_{21}(\lambda) & \hat{f}_{22}(\lambda) \end{bmatrix} \begin{bmatrix} \hat{A}(t,0) \\ \hat{B}(t,0) \end{bmatrix} = M(\lambda) \begin{bmatrix} \hat{A}(t,0) \\ \hat{B}(t,0) \end{bmatrix}, \tag{S26}$$

where $M(\lambda)$ is given by (S20). The untreated next generation operator (NGO) is therefore given by [31–33]

$$M(0) = \begin{bmatrix} M_{11}(0) & M_{12}(0) \\ M_{21}(0) & M_{22}(0) \end{bmatrix}$$

and R_0 is the spectral radius of $M(0)$. The eigenvalues of $M(0)$ are

$$\begin{aligned} \xi_{1,2} &= \frac{Tr(M(0)) \pm \sqrt{Tr(M(0))^2 - 4\det(M(0))}}{2} \\ &= \frac{M_{11} + M_{22} \pm \sqrt{(M_{11} - M_{22})^2 + 4M_{12}M_{21}}}{2}. \end{aligned}$$

In particular, we note that ξ_{12} are real numbers, and the reproductive number of the mixed population is

$$R_0 = \frac{M_{11} + M_{22} + \sqrt{(M_{11} + M_{22})^2 - 4M_{11}M_{22} + 4M_{12}M_{21}}}{2}.$$

As each individual cell can only produce a maximum of two daughter cells, we expect $R_0 \leqslant 2$. We now show that this is the case, and that this bound will be reached only if there is no death.

Lemma G. *Let the model parameters be non-negative. Then,* $0 \leqslant R_0 \leqslant 2$*, and achieves these bounds if* $r_A = r_B = 0$*, or if* $d_A = d_B = 0$ *respectively.*

Proof. The matrix $M(\lambda)$ is comprised of non-negative elements $M_{ij}(\lambda)$, so

$$0 \leqslant \frac{M_{11} + M_{22}}{2} \leqslant R_0.$$

Now, $R_0 = 0$ if and only if

$$M_{11} + M_{22} = 0 \quad \text{and} \quad (M_{11} - M_{22})^2 + 4M_{12}M_{21} = 0,$$

which can only be achieved when $r_A = r_B = 0$. To show the upper bound, we recall that

$$M_{21}(0) = \frac{2r_A}{d_A + r_A} - M_{11}(0) \quad \text{and} \quad M_{12}(0) = \frac{2r_B}{d_B + r_B} - M_{22}(0),$$

so the NGO is given by

$$M(0) = \begin{bmatrix} M_{11}(0) & \frac{2r_B}{d_B+r_B} - M_{22}(0) \\ \frac{2r_A}{d_A+r_A} - M_{11}(0) & M_{22}(0). \end{bmatrix}$$

Then, the Gershgorin circle theorem implies that

$$R_0 \in \left\{ \left[2M_{11}(0) - \frac{2r_A}{d_A + r_A}, \frac{2r_A}{d_A + r_A} \right] \bigcup \left[2M_{22}(0) - \frac{2r_B}{d_B + r_B}, \frac{2r_B}{d_B + r_B} \right] \right\},$$

so

$$R_0 \leqslant \max \left[\frac{2r_A}{d_A + r_A}, \frac{2r_B}{d_B + r_B} \right] \leqslant 2.$$

with strict inequality if both $d_A > 0$ and $d_B > 0$. Now, assume that $d_A = d_B = 0$, so that $M(0)^T$ becomes

$$M(0)^T = \begin{bmatrix} M_{11}(0) & 2 - M_{11}(0) \\ 2 - M_{22}(0) & M_{22}(0). \end{bmatrix}$$

which has spectral radius 2. Since $M(0)$ and $M(0)^T$ are similar, it follows that $R_0 = 2$ if $d_A = d_B = 0$.

□

Similar to the Malthusian parameter, the basic reproduction number is a measure of population fitness, where the sign of $R_0 - 1$ determines if cells are expected to replace themselves through replication. When the birth and death rates are balanced, we now show that we should not expect population growth.

Lemma H. *Let the model parameters be positive and assume that* $r_A - d_A = r_B - d_B = 0$. *Then* $\lambda_P = R_0 - 1 = 0$.

Proof. Since $r_A = d_A$ and $r_B = d_B$, it follows that

$$\frac{2r_A}{r_A + d_A} = \frac{2r_B}{r_B + d_B} = 1.$$

Once again using the similarity between $M(0)$ and $M(0)^T$, we compute

$$M(0)^T = \begin{bmatrix} M_{11}(0) & 1 - M_{11}(0) \\ 1 - M_{22}(0) & M_{22}(0), \end{bmatrix}$$

which clearly has spectral radius 1, so the R_0 claim in shown. Further, for $F(\lambda)$ given by (S23), it is simple to see that $F(0) = 0$ when $\lambda_A = \lambda_B = 0$. Further, Lemma C shows that $F(\lambda)$ is strictly increasing for $\lambda > \max(r_A - d_A, r_B - d_B) = 0$. Therefore, 0 is the rightmost real root of $F(\lambda)$ and $\lambda_P = 0$. □

Theorem I. *If the model parameters are positive, then, sign*$(\lambda_P) =$ *sign*$(R_0 - 1)$.

Proof. Consider the spectral radius of $M(\lambda)$ given by

$$\xi(\lambda) = \frac{M_{11}(\lambda) + M_{22}(\lambda)}{2} + \frac{\sqrt{(M_{11}(\lambda) + M_{22}(\lambda))^2 - 4M_{11}(\lambda)M_{22}(\lambda) + 4M_{12}(\lambda)M_{21}(\lambda)}}{2},$$

and note that $R_0 = \xi(0)$ while the Malthusian parameter λ_P satisfies $\xi(\lambda_P) = 1$. Viewing ξ as a function of M_{ij} for $i, j = 1, 2$, we compute

$$\begin{aligned}
\frac{\partial \xi}{\partial M_{ii}} &= \frac{1}{2} + \frac{1}{2}\left(\frac{M_{ii} - M_{jj}}{\sqrt{(M_{ii} + M_{jj})^2 - 4M_{11}M_{22} + 4M_{12}M_{21}}}\right) \\
&= \frac{1}{2} + \frac{1}{2}\left(\frac{M_{ii} - M_{jj}}{\sqrt{(M_{ii} - M_{jj})^2 + 4M_{12}M_{21}}}\right) \geqslant 0, \\
\frac{\partial \xi}{\partial M_{ij}} &= \frac{M_{ij}}{\sqrt{(M_{ii} + M_{jj})^2 - 4M_{11}M_{22} + 4M_{12}M_{21}}} \geqslant 0,
\end{aligned}$$

where we have used the non-negativity of M_{ij} to establish the sign of $\frac{\partial \xi}{\partial M_{ij}}$. Since each M_{ij} is strictly decreasing in λ, it follows that

$$\frac{\mathrm{d}\xi(\lambda)}{\mathrm{d}\lambda} = \sum_{i,j=1}^{2} \frac{\partial \xi}{\partial M_{ij}} \frac{\mathrm{d}M_{ij}}{\mathrm{d}\lambda} \leqslant 0.$$

If one of $M_{ij} \neq 0$, then the above inequality is strict, and ξ is a strictly decreasing function of λ. Moreover, $M_{ij} = 0$ for all i, j can only occur when $r_A = r_B = 0$. Therefore, for positive model parameters, $\xi(\lambda)$ is a strictly decreasing function.

Now, assume that $\lambda_P > 0$, so $R_0 = \xi(0) > \xi(\lambda_P) = 1$. Conversely, assume that $R_0 - 1 > 0$, so then $\xi(0) > 1$. As

$$\lim_{\lambda \to \infty} \xi(\lambda) = 0,$$

the intermediate value theorem ensures that $\lambda_P > 0$. Next, assume that $R_0 = \xi(0) < 1$. Then, as λ_P must exist from Theorem F, the monotonicity of ξ gives $\lambda_P < 0$. Finally, assume that $\lambda_P < 0$. It follows that $1 = \xi(\lambda_P) > \xi(0) = R_0$.

□

The sign relationship between the Malthusian paramether λ_P and the basic reproduction number R_0 established in Theorem I allows us to determine if the tumour population will grow or decay through a number of techniques. As we will show later, it is sufficient to design a treatment schedule to ensure that $R_0 < 1$, and the sign relationship established in Theorem I immediately yields that $\lambda_P < 0$ so small tumour population cannot grow. Conversely, if both $\lambda_A < 0$ and $\lambda_B < 0$, Theorem F implies that $\lambda_P < 0$, so $R_0 < 1$ from which it follows that the tumour population is decaying.

Stable age distribution and population proportion

Having calculated the Malthusian parameter, we can determine the stable age distribution. From the non-linear eigenproblem (S20), each value of the Malthusian parameter λ_P defines an eigenvector $[A_0, B_0]$ and corresponding solution to (S18) given by (S19). At a given time t, these exponential functions model the proportion of cells

born at time $t - a$ that have not died or reproduced. Perhaps counter-intuitively, a larger value of λ implies that there are fewer cells for a given age a. However, as nascent cells are necessarily born with age 0, an accumulation of old cells (large a) is indicative of a population that is not reproducing.

Finally, after solving the non-linear eigenproblem for the Malthusian parameter, we can calculate the explicit solution of (S2) under steady state growth using (S17). Then, it is simple to calculate

$$\bar{A}(t) = e^{\lambda_P t} \int_0^\infty A_0 \exp\left(-[d_A + r_A + \lambda_P]a\right) \mathrm{d}a = \frac{A_0 e^{\lambda_P t}}{d_A + r_A + \lambda}$$
$$\bar{B}(t) = e^{\lambda_P t} \int_0^\infty B_0 \exp\left(-[d_B + r_B + \lambda_P]a\right) \mathrm{d}a = \frac{B_0 e^{\lambda_P t}}{d_B + r_B + \lambda}.$$

In practice, it is difficult to calculate the age of each cell in a cohort, but relatively easy to calculate the proportion of different phenotypes. Thus, the ratio

$$\frac{\bar{A}}{\bar{A} + \bar{B}} = \frac{A_0/(d_B + r_B + \lambda_P)}{A_0/(d_B + r_B + \lambda_P) + B_0/(d_A + r_A + \lambda_P)}. \tag{S27}$$

is likely to be of clinical interest in understanding drug resistance, although the assumption of Malthusian growth is only appropriate in the case of unlimited resources. We note that this ratio is dependent on both the Malthusian parameter λ_P and the eigendirection corresponding to λ_P through $[A_0, B_0]$. Later, we will show how this ratio is dependent on the model parameters and growth function $R_I(\bar{A}(t), \bar{B}(t))$. This dependence on the growth function indicates that the ratio $\bar{A}/(\bar{A} + \bar{B})$ will evolve as a population exhausts available resources. The importance of growth phase on population make up is well established in *E.coli* populations that exhibit bet-hedging [21, 34, 35].

Frequency dependent fitness outperforms Malthusian growth

The ability to calculate the Malthusian parameter relies on the existence of a dominant exponential type solution. Exponential growth is unrealistic in the case of finite resources, as growing populations will exhaust available resources. Therefore, we also consider the dynamics of $N(t)$ for logistic and Allee type growth functions, given by (S7) and (S8) respectively. To study growth in resource rich environments, we take $N(0) \ll K$, so there are sufficient resources available initially.

In populations dominated by the "fitter" drug sensitive phenotype, it is reasonable to expect Malthusian growth to dominate resource limited growth, even in the case $N(0) \ll K$. Biologically, this corresponds to the competition for finitely many resources limiting growth, even for small populations. However, we show in Fig B that it is possible that cooperation amongst drug tolerant cells can initially out perform exponential type growth.

In Fig B, we plot the ratio of the resource limited vs unlimited growth, so Malthusian growth would correspond to a horizontal line at 1. As shown in Fig B **A**, a majority drug sensitive initial population can briefly match, or even slightly surpass (due to a slight Allee effect), Malthusian growth. As the Malthusian parameter falls between the fitnesses of phenotype A and B, a population initially comprised of exclusively drug sensitive cells will outperform Malthusian growth of the mixed population until the effects of phenotypic switching become apparent and the drug tolerant population grows in size. Moreover, as $N(t)$ increases and cells compete for limited resources, Malthusian growth overtakes and dominates the finite resource case. As drug sensitive cells are assumed to be fitter than drug tolerant cells, the presence of less fit drug tolerant cells both consumes resources and also lowers the average fitness of the population.

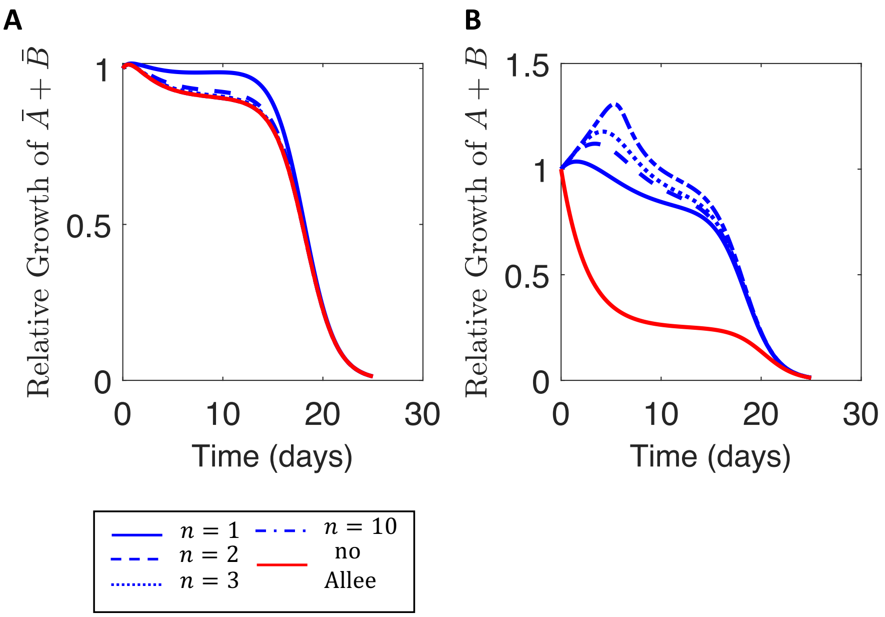

Fig B. A comparison of growth rates for different growth functions f_n, $n = 1, 2, 3, 10$, against Malthusian growth. The "no Allee" curves correspond to no frequency dependent fitness increase and $f_n = 1$. Fig **A** shows the population evolution from an initial population comprised of 100 drug sensitive cells and one drug tolerant cell for the generic parameters in Table A obtained by simulating (S15). Fig **B** shows the population evolution from an initial population comprised of 1 drug tolerant cell and 100 drug tolerant cells for the generic parameters in Table A obtained by simulating (S15).

Conversely, Fig B**B** presents a much more interesting situation. From an initial population of one drug sensitive cell and 100 drug tolerant cells, the impact of the Allee effect drastically changes initial population growth. From this high initial proportion of drug tolerant cells, the constrained growth model out performs Malthusian growth. Since the drug tolerant cells receive a substantial increase in fitness due to the Allee effect, the initial fitness of the population is higher – despite the finite amount of resources – than the fitness of the total population in the presence of unlimited resources and no cooperation. However, despite the cooperation induced increased fitness of drug tolerant cells, the population eventually evolves towards a predominantly drug sensitive population due to phenotypic switching and the growth rate falls below Malthusian growth.

Phenotype switching may mitigate fitness differences

In the main text, we showed that the proportion of drug-sensitive cells, $\bar{A}/(\bar{A}+\bar{B})$, initially decreases before reaching a plateau and remaining relatively constant as d_A is increased when resources are limited. This result indicates that the relative fitness difference between phenotypes is less important than the likelihood of phenotypic inheritance in the resource limited case. Here, we repeat these calculations with the a sufficiently large death rate of drug-sensitive cells so that $\lambda_A < 0$, simulate the model with limited resources for 500 days, and calculate the proportional of drug-sensitive cells at day 500. In Fig C, we show that, for small P_{BB}^{max}, that increasing d_A (and thus decreasing λ_A) can increase the proportion of drug-sensitive cells in the population,

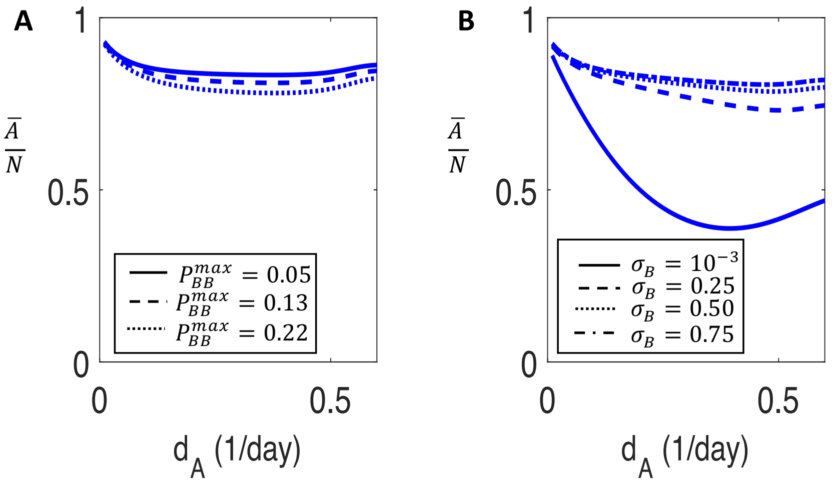

Fig C. The proportion of drug-sensitive cells for increasing values of the sensitive cell death rate. A and **B**) the proportion of drug sensitive cells in the limited-resource setting is obtained by simulating the model (S2) for 500 days and computing of drug sensitive cells at day 500. **A**) the model predictions for $P_{BB}^{max} = 0.05, 0.13, 0.22$. **B**) the model predictions for $\sigma_B = [1 \times 10^{-3}, 0.25, 0.5, 0.75]$ with $P_{BB}^{max} = 0.6$.

even if $\lambda_A < 0$.

Parameter identifiability during cancer therapy

Thus far, we have shown that the phenotypic switching strategy employed by a population can lead to different types of therapeutic resistance. Here, we discuss the difficulties of determining the switching probability based on untreated population data. We consider *in vitro* data from the growth of multiple myeloma growth in mice [36], and numerous different phenotypic switching strategies. After digitizing the data from Fig 1 **A** of [36] and fixing a phenotypic switching strategy, we fit the tumour growth parameters r_A, r_B and $d_A = d_B$ to the time series by minimizing

$$\text{Error}(r_A, r_B, d_A) = \sum_{i=1}^{n} \left(N(t_i) - \text{Data}_i\right)^2 . \tag{S28}$$

The data from [36] is sampled at times $\{t_i\}_{i=1}^n$. For a given phenotypic switching strategy and parameter set r_A, r_B and $d_A = d_B$, we simulate (S15) and sample the numerical solution at the times t_i. Equation (S28) is then the ℓ_2 distance between $N(t_i)$ and the [36] data. In addition to the switch and stay strategies discussed in the Main Text, we consider 6 additional phenotypic strategies given by the pairs $(P_{BB}^{max}, P_{BB}^{*})$: $(1, 0.25)$; $(1, 0.5)$; $(1, 0.75)$; $(0.9, 0.25)$; $(0.9, 0.5)$; and $(0.9, 0.75)$.

In Fig D **A**, we show the fitting results for each of the 8 different switching strategies to the [36] data. The eight curves are essentially indistinguishable, which indicates a possible difficulty in translating our results to the clinic. In Fig D**B**, we simulate two therapy cycles with maximal death rate $d_A^{max} = 100 d_A$, and the eight

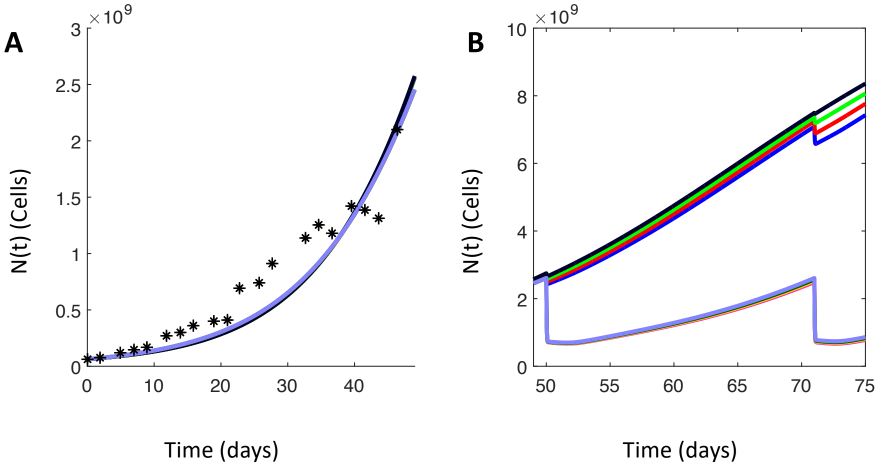

Fig D. Fitting of the mathematical model to the Dingli et al. 2009 [36] data for a variety of fitting strategies. Fig **A** shows the fitting of the mathematical model (S2) to the Dingli et al. [36] data for the 8 different switching strategies given in the text. Fig **B** shows the regrouping of same 8 strategies after 2 applications of therapy. The parameters used in these simulations are given in Table B.

Strategy	r_A (1/day)	r_B (1/day)	d_A (1/day)	P_{BB}^{max}	P_{BB}^{*}
1	0.7101	0.6745	0.6255	1	0.25
2	0.6987	0.6637	0.6144	1	0.50
3	0.7008	0.6658	0.6167	1	0.75
4	0.6956	0.6608	0.6116	1	0.95
5	0.6969	0.6089	0.5968	0.9	0.0
6	0.6973	0.6093	0.5971	0.9	0.25
7	0.6989	0.6107	0.5985	0.9	0.5
8	0.6999	0.6115	0.5992	0.9	0.75

Table B. The tumour growth parameters obtained by fitting (S2) **to the Dingli et al. [36] data for the 8 different switching strategies.**

previously indistinguishable curves are then separated into two clusters almost immediately following therapy. The four strategies corresponding to the less responsive (larger $N(t)$: black, green, red and blue) curves all have $P_{BB}^{max} = 1$, while the four strategies corresponding to higher response to therapy (lower $N(t)$: purple and other colors) all have $P_{BB}^{max} = 0.9$. Thus, we see that population response to treatment can stratify populations by their switching strategy. Therefore, it should be possible to use treatment response to determine the approximate switching strategy of a tumour biopsy and use this information to inform therapeutic strategies.

Generic strategy to avoid treatment failure

For the generic tumour growth parameters used thus far, $r_B/r_A = 1/2$. While our results are robust to different values of ε, we illustrate our results with $\varepsilon = 0.7$ in the threshold ratio ϑ^* from the Main Text Eq. (5) . To test if this simple threshold ratio is sufficient to avoid the establishment of therapy, we follow the same periodic dosing as shown in Main Text Fig 3, but we only administer therapy if the ratio $\bar{B}(t)/\bar{A}(t) < \vartheta^*$ and label this strategy *adjustable therapy*.

However, it is unrealistic that clinicians will determine the ratio $\bar{B}(t)/\bar{A}(t)$ and immediately administer therapy. Therefore, to decide if treatment will be applied at

time t^*, we consider $\bar{B}(t^*-1)/\bar{A}(t^*-1) < \theta^*$ which corresponds to clinicians taking one day to complete the phenotype profile of the tumour. We show that adjustable therapy avoids the establishment of resistance in Fig E. As in the Zhang et al. [22] trial, the main benefit of this adjustable therapy is, that by avoiding the development of resistance, therapy with the same drug can continue indefinitely. In particular, the effectiveness of the *adjustable therapy*, as measured by disease burden (S29), increases the longer that therapy is applied. We note that, rather than using the ratio of drug tolerant to sensitive cells to determine if therapy should be applied, it is possible to use a cancer specific biomarker [22].

Inspired from the success of adaptive therapy in prostate cancer [16, 22], we developed a simple strategy to avoid the establishment of resistance in *Avoiding the establishment of a drug tolerant population*. We show the results from that section here. It is important to note, once again, that our model is quite coarse, so these results serve more as a proof-of-concept, rather than a proposed therapeutic strategy. We measure treatment efficiency by

$$\text{Burden}(S) = \frac{1}{T}\int_0^T \frac{N(\tau)}{K}\mathrm{d}\tau = \frac{1}{T}\int_0^T \frac{\bar{A}(\tau)+\bar{B}(\tau)}{K}\mathrm{d}\tau. \tag{S29}$$

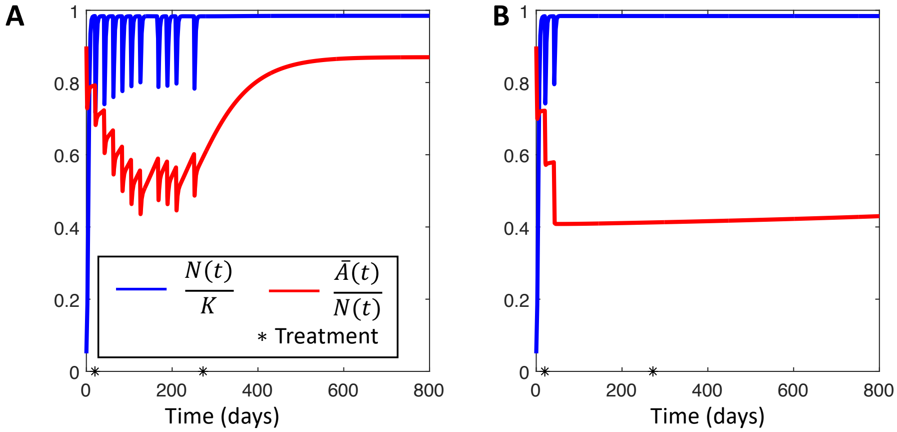

Fig E. The effect of adjustable therapy on a population using either a *switch* or *stay* strategy. The *switch* population is shown in Figure **A**) and the *stay* population is shown in Figure **B**). In both cases, treatment is applied between the black stars on days 20 and 272. The red curve shows the proportion of drug sensitive cells $\bar{A}(t)/N(t)$ and the blue curve shows the dynamics of $N(t)$. The parameters used in these simulations are given in Table A.

Figure E demonstrates that the proposed adjustable therapy strategies avoid the establishment of a dominant resistant phenotype. We compare the results of the adaptive therapy against periodic treatment in Fig E. Fig E **A** shows the response of a population with a switch strategy ($P_{BB}^{max} = 0.9, P_{BB}^* = 0$) and transient resistance to adjustable therapy, while Fig E **B** shows the same effect of adjustable therapy on a population with a stay strategy and permanent resistance ($P_{BB}^{max} = 1, P_{BB}^* = 0.95$). When comparing the effectiveness of the therapy using (S29) over 240 days of treatment, the adjustable therapy is leads to a disease burden that is 0.2% lower (for the switch strategy) or 0.03% higher (for the stay strategy) than the periodic therapy in Fig E **A** and **B**, respectively.

If these adjustable strategies are continued for longer than 240 days, there is an increasingly important decrease in disease burden when compared to the periodic type treatment resulting from the eventual ineffectiveness of the periodic dosing due to

therapeutic resistance that occurs when the population is dominated by the resistant phenotype.

General results are robust to parameter variation

We have thus far shown that changes in phenotypic switching strategy can lead to two distinct types of therapy resistance for a given parameter set. Here, we confirm that this qualitative result is robust to parameter variations, and that the results presented in the main text are representative of model dynamics for a number of different parametrizations. We continue to enforce $d_A = d_B$ and explore the possible parameter combinations by selecting triplets of parameter values from the set

$$\tilde{r}_A \in \{0.7, 1.4, 0.4\} \quad \tilde{r}_B \in \{0.35, 0.1, 0.035\} \quad \sigma_{A,B} \in \left\{1 \times 10^{-1}, 1 \times 10^{-2}, 1 \times 10^{-3}\right\}. \tag{S30}$$

For a given parameter combination, we simulated the mathematical model with periodic treatment as described previously.

Each of the 27 possible parameter combinations were tested with both the switch and stay strategies for the drug tolerant population and each demonstrated transient resistance and the re-establishment of the wild type phenotype shortly after cessation of therapy when coupled with a switch strategy, similar to Main Text Fig 3**A**. Conversely, when simulated with a stay strategy, each parameter combination demonstrated the permanent resistance shown in Main Text Fig 3**B**. Therefore, we consider the simulations shown to be representative.

As demonstrated, the switch and stay strategies are crucial in determining the appearance and duration of therapeutic resistance. However, the probabilities P_{BB}^{max} and P_{BB}^{*} used to determine the switch and stay strategies are vastly different. Therefore, we test the robustness of the qualitative results shown in Main Text Fig 3 for different extremes of the switch and stay strategies. Once again, we consider two distinct strategies. In the first, we fix $P_{BB}^{max} = 0.9$, as in the switch strategy, and test different values of P_{BB}^{*} from $P_{BB}^{*} \in \{0, 0.25, 0.5, 0.75, 0.9\}$. Then, for each value of P_{BB}^{*}, we simulate periodic therapy. For $P_{BB}^{*} \in \{0, 0.25, 0.5, 0.75\}$, resistance was transient and the wild type phenotype was re-established in the population shortly after the end of therapy. Conversely, for $P_{BB}^{*} = P_{BB}^{max} = 0.9$, re-establishment of the wild type population took over 800 days after treatment cessation, which is effectively permanent resistance. We note that, if $P_{BB}^{max} = P_{BB}^{*} = 0.9$, then reproductive resistant cells are quite likely to transfer their phenotype to offspring, and this strategy closely resembles a staying strategy. As a consequence, the correspondence between transient resistance and a “switch” strategy appears to be robust to parameter changes.

Now, to test if a stay strategy consistently predicts permanent resistance, we fix the homoeostatic switching probability $P_{BB}^{*} = 0.9$ and vary $P_{BB}^{max} \in \{0.92, 0.94, 0.96, 0.98, 1\}$. For all values of P_{BB}^{max}, periodic therapy led to resistant phenotype dominance that persisted for over 800 days following the end of therapy. Once again, in the context of therapeutic scheduling, this resistance is effectively permanent. Thus, the stay strategy consistently leads to permanent treatment resistance, and we consider our results in the Main Text to be representative.

Effectiveness of Adjustable Therapy

We also verified the effectiveness of adjustable therapy for the combination of growth parameters given in (S30). Once again, we tested all 27 possible combinations of parameters in (S30) for both the switch and stay strategy, while holding the measure of acquired fitness, ε, fixed at $\varepsilon = 0.7$. For the switch strategy, adjustable therapy improved upon periodic therapy in 15/27 cases, with 8 of the remaining cases showing

less than a 0.0001% increase in tumour burden. In the worst case, adjustable therapy led to an increase in tumour burden by 3%, while in the best case, there was a 2% decrease.

We completed the same test for a population with a stay strategy. There, adjustable therapy improved upon periodic therapy in 15/27 cases. In the worst case, adjustable therapy led to an increase in tumour burden by 0.6%, while in the best case, there was a 0.2% decrease. Therefore, the therapeutic improvement offered by adjustable therapy appears to be robust to parameter variations. Moreover, the adjustable schedule consistently avoided the establishment of resistance.

These results differ from the sustained treatment success in for NSCLC in the main text for an important reason, namely that, for the generic parametrization considered here, $\lambda_B > 0$. Accordingly, our analysis and derivation of the model informed therapy in the main text does not apply. Therefore we are not using an optimized dose size, and as the drug tolerant cells are entirely resistant to therapy, it is not possible to drive $R_0^* < 1$.

Implementation of the model in Matlab

In general, we simulated the system of ODEs (S15) with the given initial conditions using the 4th order adaptive Runge-Kutta solver ODE45 in Matlab [37] with relative and absolute error tolerances of 1×10^{-6}. We implemented the chemotherapeutic dosing as a time-dependent indicator function of the set $(t_i, t_i + T_{admin})$ as written in (S10).

We fit the *in vitro* data using the Matlab function FMINCON with 15 distinct initial points. To evaluate the objective function given by the ℓ_2 norm between the model simulations and observed data, we simulated the model using the ODE45 solver for each parameter set. We then computed the total number of cells, given by $N(t)$, in the numerical solution of the differential equation at the data sampling time points. To evaluate the objective function for each candidate set of parameter values, we computed the sum of squares difference between the model simulation and the observed data.

To calculate the Malthusian parameter, we fixed $r_A = r_B = 0.7$ and varied $d_{A,B} \in [0.0105, 1.05]$. We took 100 evenly spaced samples from the interval $[0.0105, 1.05]$ for both d_A and d_B. For these 10 000 pairs, we solved the implicit equation (S23) for λ_P using the Matlab script fzero with 5 initial points evenly spaced between λ_A and λ_B. During Malthusian growth, the ratio of the stable age distributions is determined by (S27) where λ_P is the Malthusian parameter while A_0 and B_0 are the entries in the eigenvector corresponding to eigenvalue 1 of (S20). After calculating the Malthusian parameter and the corresponding entries of (S20) for $\lambda = \lambda_P$, we found the corresponding eigenvector using the Matlab function eigs. When considering population composition under limited resources, we simulated the model using ODE45 for 500 days to ensure convergence to equilibrium and calculated $A(500)/N(500)$ directly from the numerical simulation.

Application to non-small cell lung cancer

Similar to the experiments that identified persisters in bacterial populations [35,38], the experimental set up used by Craig et al. [39] begins with a constant environment and a genotypically homogeneous population of cancer cells. those experiments, Craig et al. [39] cloned a cell line with an oncogenic Kras, homozygous p53 and heterozygous Dicer1 loss of function mutations that induces tumours when injected into mice. Growth of non-small cell lung cancer (NSCLC) tumour spheroids was quantified as previously described [39,40]. The parental (WT) cell line was derived from KRas-G12D, p53−/−, Dicer1f/− genotype lung tumours and mutants (M1 and M2) were obtained through transfection to Dicer1+/+ and Dicer1-/- using CRISPR-Cas9 [40]. It may therefore be tempting to conclude that the phenotypic heterogeneity present is solely

Cell Type	Drug	P_{BB}^{max}	P_{BB}^{*}	$\sigma_A = \sigma_B$ (1/hour)	d_A^{max} (1/hour)
WT	Docetaxel	0.93	0.62	1×10^{-2}	0.7025
WT	Afatinib	0.95	0.81	1×10^{-2}	0.4828
WT	Bortemozib	0.95	0	1×10^{-2}	0.7993
M1	Docetaxel	0.91	0	1×10^{-2}	1.145
M1	Afatinib	0.92	0	1×10^{-2}	1.699
M1	Bortemozib	92	0	1×10^{-2}	0.4491
M2	Docetaxel	0.71	0.09	1×10^{-2}	0.1447

Table C. The switching parameters for WT, M1, and M2 cell lines obtained by fitting (S2) to the tumour growth data.

due to stochastic phenotype switching. However, the distinction between phenotypic plasticity, wherein cells change phenotype in response to environmental change, and truly stochastic phenotype switching is subtle [38]. Moreover, this dichotomic representation of phenotypic heterogeneity does not account for partially heritable phenotype, as reported by Yang et al. [41] and considered in our model. Nevertheless, the NSCLC data offers an initial opportunity to apply our simple mathematical model to cancer data and explore the role of phenotype switching in treatment resistance.

Parameter fitting

Here, we present the results of the fitting procedure for the WT, M1 and M2 data from [39]. As mentioned in the Main Text, we fit this data by minimizing the ℓ_2 error between the data and the model simulation. We used the algorithm *fmincon* [37] with 15 distinct initial points. We show the results of the fitting for the WT, M1 and M2 data in Figs F and G, and give the parameter values in Table C and D. As the data is collected on a short time frame, we give the parameter estimates in units of 1/hour, but convert to 1/day when performing longer term calculations.

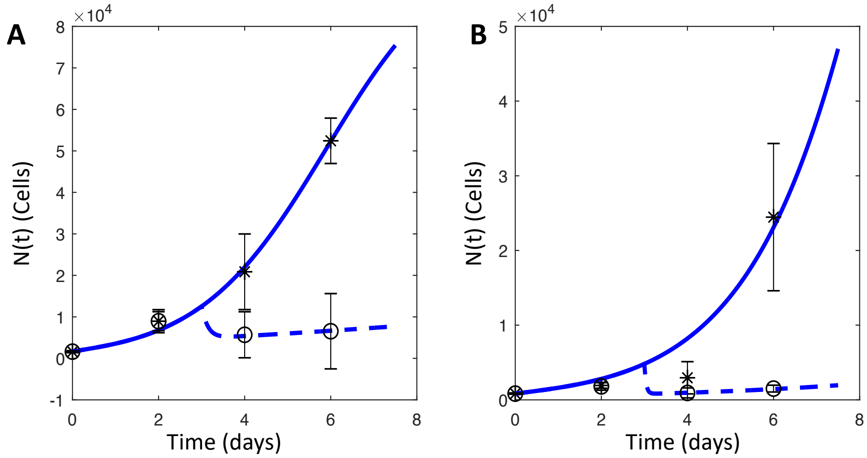

Fig F. Fitting results of Equation (S2) **to the WT and M1 data from [39] treated with docetaxel.** The model fits to the WT and M1 data are shown in Figs **A** and **B**, respectively. In all cases, the untreated data is given by the black stars while the untreated simulation is in solid blue. The docetaxel treated data is given by the hollow circles and the treated simulation is in dashed blue. The parameters used in these simulations are given in Tables C and D.

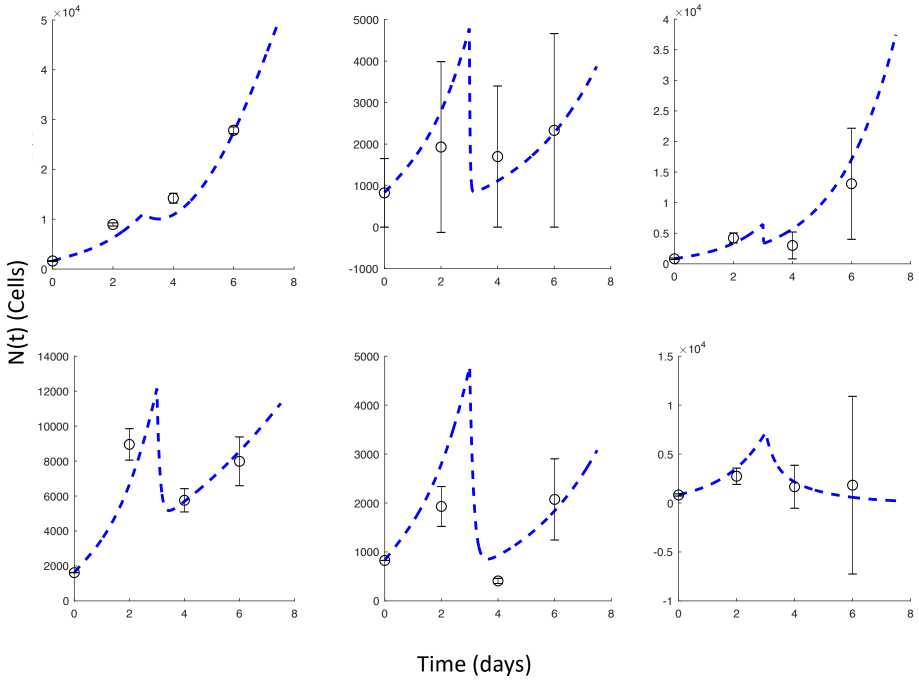

Fig G. Fitting results of Equation (S2) **to the WT, M1 and M2 data from** [39] **treated with afatinib and bortezomib.** The top row shows the model fits to the WT, M1, and M2 data treated with afatinib, respectively. The bottom row shows the model fits to the WT, M1, and M2 data treated with bortezomib, respectively. The parameters used in these simulations are given in Tables C and D.

Cell Type	r_A (1/hour)	r_B (1/hour)	d_A (1/hour)
WT	0.4827	0.3498	0.4198
M1	0.1646	0.0100	0.1163
M2	0.1114	0.0857	0.0768

Table D. The tumour growth parameters for the WT, M1 and M2 type cells obtained by fitting (S2) to the tumour growth data.

Treatment induced periodic environment

Most anti-cancer therapies include a recovery period following each treatment where the drug washes out and the patient recovers from the effects of treatment. Classical chemotherapy induces an approximately periodic tumour microenvironment with respect to the concentration of the chemotherapeutic agent, where each treatment cycle acts as the beginning of a new period. In what follows, we assume that the chemotherapeutic drug $C(t)$ is administered periodically with a period of T days, and eliminated according to (S10) with elimination rate k_{elim}. To facilitate the calculation of the reproduction number during treatment, we derive an estimate for the forced limit cycle in $C(t)$ during periodic therapy.

Now, let the first dose be given at time $t = t_0$, and assume that the administration time, T_{admin} is negligible, so that each administration of therapy is given as an impulse at time $t_0 + nT$. Then, for $t \in (t_0, t_0 + T)$, $C(t) = \frac{Dose}{Vol} \exp(-k_{elim}(t - t_0))$. At time $t = T$, a second dose is given, so $C(T) = \frac{Dose}{Vol}[1 + \exp(-k_{elim}(T - t_0))]$, and the concentration of the drug decays, for $t \in (T, 2T)$, according to $C(t) = C(T) \exp(-k_{elim}(t - T))$. Proceeding inductively, we see that, immediately after

administering the $n+1$st dose at $t = t_0 + nT^+$,

$$C(t_0 + nT^+) = \frac{Dose}{Vol} \sum_{m=0}^{n} \exp(-k_{elim} mT) = \frac{Dose}{Vol} \left(\frac{1 - \exp(-k_{elim}(n+1)T)}{1 - \exp(-k_{elim}T)} \right).$$

Then, for $t \in (t_0 + nT, t_0 + (n+1)T)$, $C(t) = C(nT^+) \exp(-k_{elim}(t - NT))$. As the number of administrations, n, grows, the term $\exp(-k_{elim}(n+1)T)$ becomes increasingly small. Recalling that the half effect of the chemotherapeutic is given by $C_{1/2}$, we discard the influence of drug concentrations that are less than $\omega C_{1/2}$ for a given value of $\omega \ll 1$. Thus, after

$$N_\omega = \left\lceil \frac{\log\left(\omega C_{1/2} Vol/Dose\right)}{-k_{elim}T} \right\rceil - 1,$$

administrations, the error induced by discarding the $\exp(-k_{elim}(N_\omega + 1)T)$ terms is

$$\left(\frac{Dose}{Vol}\right) \left| \frac{1 - \exp(-k_{elim}(N_\omega + 1)T)}{1 - \exp(-k_{elim}T)} - \frac{1}{1 - \exp(-k_{elim}T)} \right| \leqslant \frac{\omega C_{1/2}}{1 - \exp(-k_{elim}T)}.$$

Accordingly, for a given value of ω and after the N_ω-st administration of the chemotherapeutic, we consider the drug concentration in the tumour microenvironment to be in a periodically forced limit cycle given by

$$C(t) = \left(\frac{Dose}{Vol}\right) \left(\frac{1}{1 - \exp(-k_{elim}T)}\right) \exp(-k_{elim}(t \mod T)). \tag{S31}$$

R_0^* in the treated environment

Having derived an estimate for the chemotherapeutic concentration during metronomic therapy, we study the effect of this therapy on tumour growth. Once again, we are assuming that the drug tolerant population is not self-sustaining and has a negative intrinsic growth rate, $\lambda_B < 0$, as in the NSCLC data considered. We assume that the chemotherapeutic has been administered at least N_ω times so that the tumour microenvironment is roughly periodic and consider the age structured PDE

$$\left.\begin{aligned} \partial_t A(t,a) + \partial_a A(t,a) &= -[d_A(t) + R_A(\bar{A}(t), \bar{B}(t))]A(t,a) \\ \partial_t B(t,a) + \partial_a B(t,a) &= -[d_B + R_B(\bar{A}(t), \bar{B}(t))]B(t,a), \end{aligned}\right\} \tag{S32}$$

As in our calculation of the stable age distribution, we once again consider the linearised version of (S32) corresponding to Malthusian growth. Now, solving (S32) along the characteristic lines gives

$$\begin{aligned} A(t,a) &= A(t-a,0) \exp\left[-\int_{t-a}^{t} d_A(s) + r_A \mathrm{d}s \right] \\ B(t,a) &= B(t-a,0) \exp\left[-(d_B + r_B)a \right]. \end{aligned}$$

We thus obtain the corresponding renewal equations

$$\begin{aligned} A(t,0) &= \int_0^\infty 2r_A \beta_{AA}(a) \exp\left[-\int_{t-a}^{t} d_A(s) + r_A \mathrm{d}s \right] A(t-a,0) \mathrm{d}a \\ &\quad + \int_0^\infty 2r_B (1 - \beta_{BB}(a)) \exp\left[-(d_B + r_B)a \right] B(t-a,0) \mathrm{d}a \\ B(t,0) &= \int_0^\infty 2r_A (1 - \beta_{AA}(a)) \exp\left[-\int_{t-a}^{t} d_A(s) + r_A \mathrm{d}s \right] A(t-a,0) \mathrm{d}a \\ &\quad + \int_0^\infty 2r_B \beta_{BB}(a) \exp\left[-(d_B + r_B)a \right] B(t-a,0) \mathrm{d}a. \end{aligned}$$

Taking the Laplace transform of these renewal equations gives the linear system

$$\left[\begin{array}{c}\hat{A}(t,0)\\ \hat{B}(t,0)\end{array}\right]=\left[\begin{array}{cc}\hat{f}^*_{11}(\lambda) & \hat{f}^*_{12}(\lambda)\\ \hat{f}^*_{21}(\lambda) & \hat{f}^*_{22}(\lambda)\end{array}\right]\left[\begin{array}{c}\hat{A}(t,0)\\ \hat{B}(t,0)\end{array}\right]=\hat{F}(\lambda)\left[\begin{array}{c}\hat{A}(t,0)\\ \hat{B}(t,0)\end{array}\right],$$

The treated NGO is therefore time dependent, due to the drug induced periodicity in the tumour microenvironment, and given by

$$(K_T h)(t)=\int_0^\infty \Psi(t,a)h(t-a)\mathrm{d}a,$$

where, recalling that $\beta_{ij}(a)=1-\beta_{ii}(a)$,

$$\Psi(t,a)=\left[\begin{array}{cc} 2r_A\beta_{AA}(a)\exp\left[-\int_{t-a}^t d_A(s)+r_A\mathrm{d}s\right] & 2r_B\beta_{BA}(a)\exp\left[-(d_B+r_B)a\right]\\ 2r_A\beta_{AB}(a)\exp\left[-\int_{t-a}^t d_A(s)+r_A\mathrm{d}s\right] & 2r_B\beta_{BB}(a)\exp\left[-(d_B+r_B)a\right]\end{array}\right].$$

Now, it is important to note that periodic therapy does not immediately induce a periodic environment. However, as shown, for a large number of administrations, the error induced by assuming that $C(t)$ is given by (S31) and can be made arbitrarily small. Moreover, we are interested in the asymptotic behaviour of the population of the tumour population, and therefore make the simplifying assumption that the drug concentration, and thus the environment, is effectively periodic.

The treated NGO K_T acts on the space of T–periodic functions $\mathcal{C}_T(\mathbb{R};\mathbb{R}^2)$. However, this is inconvenient for calculation purposes and we follow [28, 42] and pass the periodicity from the function h to the operator by defining

$$(\hat{K}_T g)(t)=\int_0^T \Theta_T(t,\sigma)g(\sigma)\mathrm{d}\sigma$$

where $\hat{K}_T$ acts on $\mathcal{C}((0,T);\mathbb{R}^2)$, and $\Theta_T(t,\sigma)$ is a periodic function defined by

$$\Theta_T(t,\sigma)=\begin{cases}\sum_{n=0}^\infty \Psi(t,\sigma+nT) & \text{if} \quad t\geqslant\sigma\\ \sum_{n=1}^\infty \Psi(t,\sigma+nT) & \text{if} \quad t<\sigma.\end{cases}$$

It follows that the spectral radius of K_T equals that of $\hat{K}_T$ [28, 42]. After interchanging the order of integration and summation, the treated basic reproduction number R_0^* is given by the spectral radius of

$$\mathcal{M}=\left[\begin{array}{cc}\mathcal{M}_{11} & \mathcal{M}_{12}\\ \mathcal{M}_{21} & \mathcal{M}_{22}\end{array}\right],$$

where

$$\mathcal{M}_{11}=\sum_{n=0}^\infty\int_0^T 2r_A\beta_{AA}(a+nT)\exp\left[-\int_{t-a-nT}^t d_A(s)+r_A\mathrm{d}s\right]\mathrm{d}a$$

$$\mathcal{M}_{12}=\sum_{n=0}^\infty\int_0^T 2r_B(1-\beta_{BB}(a+nT))\exp\left[-(d_B+r_B)(a+nT)\right]\mathrm{d}a$$

$$\mathcal{M}_{21}=\sum_{n=0}^\infty\int_0^T 2r_A(1-\beta_{AA}(a+nT))\exp\left[-\int_{t-a-nT}^t d_A(s)+r_A\mathrm{d}s\right]\mathrm{d}a$$

$$\mathcal{M}_{22}=\sum_{n=0}^\infty\int_0^T 2r_B\beta_{BB}(a+nT)\exp\left[-(d_B+r_B)(a+nT)\right]\mathrm{d}a$$

As the drug tolerant cells are immune to therapy, both $\mathcal{M}_{12}$ and $\mathcal{M}_{22}$ are constant in time, so these infinite series telescope and we immediately see $M_{12} = \mathcal{M}_{12}$ and $M_{22} = \mathcal{M}_{22}$, where M_{i2} is defined in the untreated NGO. It remains to calculate the effects of therapy on $\mathcal{M}_{11}$ and $\mathcal{M}_{21}$. Treatment increases the death rate of drug susceptible cells via

$$\hat{d}_A(t,a) = d_A a + (d_A^{max} - d_A)\int_{t-a}^{t} \frac{C(s)}{C(s)+C_{1/2}} \mathrm{d}s,$$

Using the estimate for $C(t)$ during periodic therapy derived previously, it is possible to calculate $\hat{d}_A(t,a)$ explicitly, and thus calculate R_0^*. However, we recall that $R_0 = 1$ is the threshold between disease growth and decay, and therefore the value of clinical interest. We now calculate a relationship between dose frequency, T and dose size to ensure that the treated reproduction number is below this threshold, so $R_0^* < 1$. Accordingly, if

$$\frac{r_A - d_A}{d_A^{max} - d_A} = \frac{\lambda_A}{d_A^{max} - d_A} < \frac{\int_{t-a}^{t} \frac{C(s)}{C(s)+C_{1/2}} \mathrm{d}s}{a}, \tag{S33}$$

it follows that

$$\exp\left[-\int_{t-a}^{t} d_A(s) + r_A \mathrm{d}s\right] < \exp\left[-2r_A a\right] \quad \text{and} \quad \mathcal{M}_{11} < 1.$$

Then, we see that

$$\begin{aligned}\mathcal{M}_{21} &= \sum_{n=0}^{\infty}\int_0^T 2r_A(1-\beta_{AA}(a+nT))\exp\left[-\int_{t-a-nT}^{t} d_A(s) + r_A \mathrm{d}s\right]\mathrm{d}a \\ &< \left[\sum_{n=0}^{\infty}\int_0^T 2r_A \exp\left[-2r_A(a+nT)\mathrm{d}s\right]\mathrm{d}a\right] - \mathcal{M}_{11} = 1 - \mathcal{M}_{11}.\end{aligned}$$

From the proof of Theorem I, R_0^* is a strictly increasing function of $\mathcal{M}_{21}$, so it follows that

$$R_0^* < \rho(\hat{\mathcal{M}})$$

where $\rho(\hat{\mathcal{M}})$ is the spectral radius of

$$\hat{\mathcal{M}} = \begin{bmatrix} \mathcal{M}_{11} & \mathcal{M}_{12} \\ 1-\mathcal{M}_{11} & \mathcal{M}_{22} \end{bmatrix}.$$

Using the fact that $\lambda_B < 0$, the Gershgorin Circle Theorem gives

$$R_0^* < \rho(\hat{\mathcal{M}}) < \max\left[1, \frac{2r_B}{r_B + d_B}\right] \leqslant 1.$$

Thus, the condition (S33) is sufficient to ensure that $R_0^* < 1$. We now use the estimate for the periodic chemotherapeutic concentration derived in (S31) to derive a dose size to ensure that (S33) holds and, more importantly, that $R_0^* < 1$. For notational simplicity, denote $\alpha_T = \left(\frac{Dose}{Vol}\right)\left(\frac{1}{1-\exp(-k_{elim}T)}\right)$ so

$$C(t) \in [\alpha_T \exp(-k_{elim}T), \alpha_T].$$

Now, let C^* be the solution of

$$\frac{\lambda_A}{d_A^{max} - d_A} = \frac{C^*}{C^* + C_{1/2}},$$

so

$$C^* = C_{1/2}\frac{\lambda_A}{-\lambda_A^*},$$

and a sufficient condition for (S33) to hold is

$$C^* < \alpha_T e^{-k_{elim}T}. \tag{S34}$$

To see that (S34) is necessary for (S33) to hold, first note that if the required dose C^* satisfies $C^* > \alpha_T$, then it is not possible to administer a large enough dose to drive $R_0^* < 1$ and (S33) cannot hold. Now, consider the case where that $\alpha_T > C^* > \alpha_T e^{-k_{elim}T}$. As $C(t)$ attains the lower bound $C = \alpha_T e^{-k_{elim}T}$ directly before the subsequent administration, it follows from the assumption $C^* > \alpha_T e^{-k_{elim}T}$ that there exists t^* such that $C(t^*) < C^*$. As $C(t)$ is continuous and $\alpha_T > C^*$, there must be an a^* such that $C(t^* - a^*) = C^*$. Then, since $C(t)$ is strictly decreasing between drug administrations, it follows that $C(s) < C^*$ for $s \in (t^* - a^*, t^*)$ and

$$\frac{\int_{t^*-a^*}^{t^*} \frac{C(s)}{C(s)+C_{1/2}}\mathrm{d}s}{a^*} < \frac{\int_{t^*-a^*}^{t^*} \frac{C^*}{C^*+C_{1/2}}\mathrm{d}s}{a^*} = \frac{\lambda_A}{d_A^{max} - d_A}.$$

Consequently, $R_0^* > 1$ during the interval $(t^* - a^*, t^*)$ for each administration period, and the tumour population may not decay. Thus, we conclude that the dose size must be chosen such that (S34) holds, which gives the threshold dose size

$$\frac{\lambda_A}{-\lambda_A^*}\alpha_T = Dose^*. \tag{S35}$$

For a given chemotherapeutic agent, both $C_{1/2}$ and k_{elim} are fixed. Thus, the attending clinician can control the quantity $e^{-k_{elim}T}/C_{1/2}$ by varying treatment frequency and intensity.

Limiting cooperation of drug tolerant cells

It may be tempting to increase the amount of chemotherapeutic administered, and indeed (S35) appears to supports the usage of maximally tolerated dosing of anti-cancer drugs. However, this maximal dose size may allow for the competitive release of a drug-tolerant phenotype and the resulting resistance to therapy, which was not considered in the preceding analysis. Thus, therapy must be designed to balance the need to ensure $R_0^* < 1$ while guarding against the evolution of resistance. We incorporated the Allee effect and cooperation of drug tolerant cells in the mathematical model through the function $f_n(\theta)$. Naively including cooperation, the fitness of the drug tolerant population is given by

$$r_B f_n(\theta) - d_A = r_B - d_A + (r_A - r_B)\frac{\theta^n}{\theta^n + 1}.$$

Thus, the threshold ratio θ^* must ensure $r_B f_n(\theta) - d_A < 0$. Using the definition of $f_n(\theta)$ and re-arranging gives

$$\frac{\theta^n}{\theta^n + 1} < \frac{-\lambda_B}{r_A - r_B} = \frac{-\lambda_B}{\lambda_A - \lambda_B}.$$

Then, a simple calculation gives the ratio θ^* defined in the main text.

Parametrization of chemotherapeutic pharmacokinetics

Docetaxel has an effective half life of roughly 86 hours [43], so we set $k_{elim} = \log(2)/86/24$ days^{-1} in our simple pharmacokinetic model, and an *in vitro* half-effect concentration of 4ng/mL [44], which is orders of magnitude less than the achievable plasma concentrations. Using the common dose size of 100mg/m^2, and volume of distribution 74L/m^2 [45], the ratio of Dose/Vol for half-maximum effect is roughly 10^4. We used the d_A^{max} value calculated from the parameter fitting in the Methods to complete the pharmacodynamic model of docetaxel. To simulate the fixed therapy schedule, we set $T = 7$ days and fixed $C_{1/2} = 0.5$. For $Vol = 74$L/m^2, the dose size during the fixed therapy schedule was calculated by satisfying Dose/$Vol/C_{1/2} = 10^4$. As previously mentioned, it is this ratio and the value of d_A^{max} that determines the pharmacodynamics in our simple model.

To simulate the administration of afatinib, we set $t_{1/2} = 37$ hours [46,47] . Afatinib is administered daily as an 40 mg oral capsule with $C_{max} = 25.2$ng/mL [46,47]. The steady state concentration of daily afatinib is roughly $2.11 \times C_{max}$ [47]. As we do not update the model for $C(t)$ to be specific for oral administration of drug, we can use the approximation for the steady state concentration of $C(t)$ to calculate

$$2.11 \times C_{max} = \frac{\text{Dose}/Vol}{C_{1/2}} \left(\frac{1}{1 - e^{-k_{elim} T}} \right).$$

Then, once again we can calculate the ratio of Dose/$C_{1/2}$, which along with the elimination constant k_{elim} determines the pharmacokinetics and pharmacodynamics of afatinib.

To simulate the fixed periodic administration of bortezomib, we set $t_{1/2} = 40$ hours, and take $T = 3$ days to account for the minimum 72 hours between intravenous administrations [48]. Bortezomib has a large volume of distribution, between 498–1884 L/m^2, so we take $Vol = 850$ L, while [48] report $C_{max} = 162$ ng/mL following multiple I.V administrations. Thus, we once again use the approximation for the steady state concentration of $C(t)$ to calculate the value of Dose.

Model informed therapy for other therapeutics

We implemented the model informed therapeutic strategy for afatinib and bortemozib in the WT and M1 cells and show the results in Figs H and I. We note that the parameter estimates for the M2 population do not satisfy $r_B < d_B$, so the model informed therapy cannot be applied. For WT and M1 treated with afatinib and bortezomib, the increased effectiveness of the model informed therapy over 100 days of therapy is shown in Table E.

We note that the model informed therapy for WT cells with afatinib does not lead to population extinction. For WT cells treated with afatinib, $r_A - d_A^{max} = -6.0842 \times 10^{-05}$. It follows that it must be the case that $C(t) \gg C_{1/2}$ if (S34) is to be satisfied. Accordingly, $C(t)$ decays too slowly between doses to inhibit the establishment of a drug tolerant population. This is illustrated in Figs G**B** and **D**. Thus, the model informed therapy, while outperforming periodic dosing, does not drive tumour extinction.

Cell Type	Drug	Treatment Effectiveness
WT	Afatinib	0.9587
WT	Bortemozib	0.7640
M1	Afatinib	0.4308
M1	Bortemozib	0.4397

Table E. The effectiveness of model informed therapy when compared to periodic dosing over 150 days of therapy

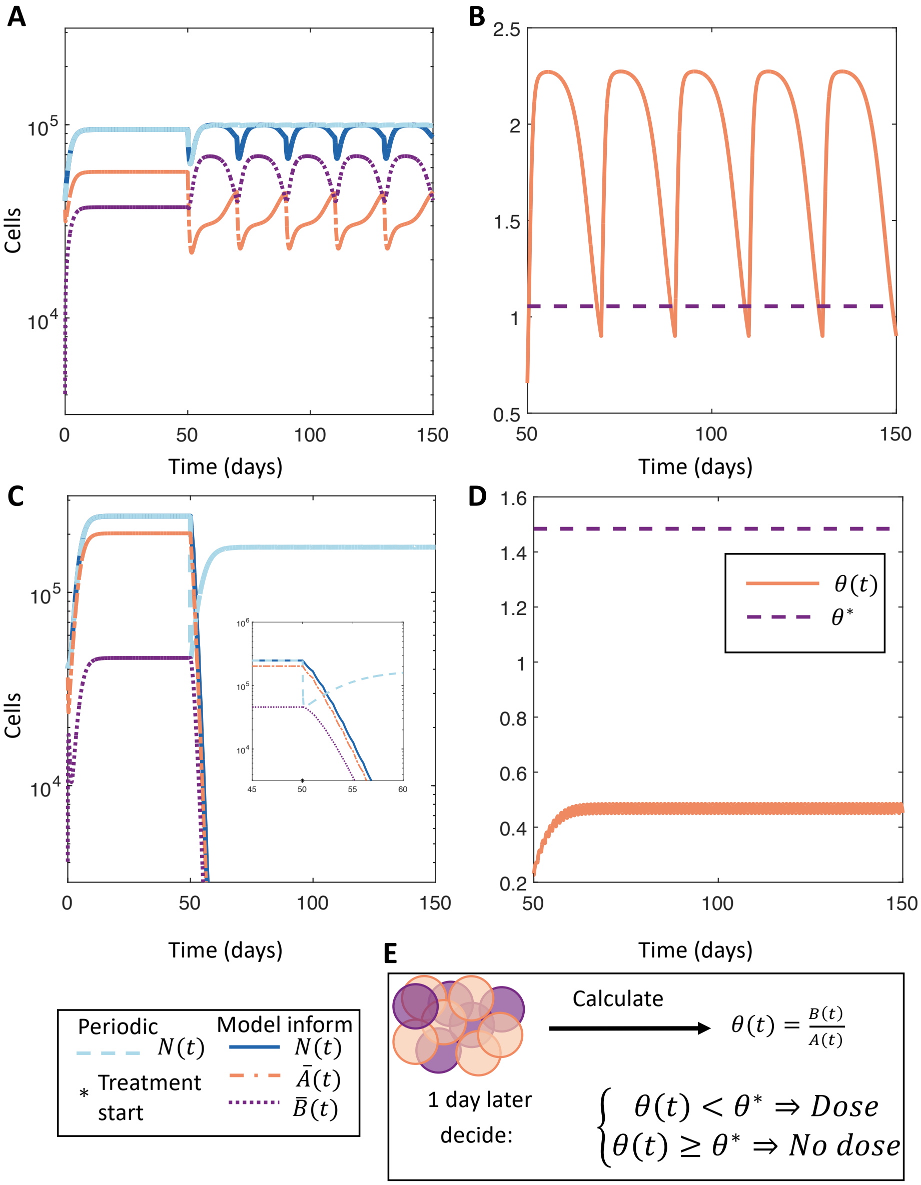

Fig H. Comparing model informed therapy and periodic dosing for afatinib. Figures **A** and **C** compare model-informed therapeutic strategy with $T = 3$ against periodic treatment with afatinib for the WT and M1 populations, respectively. The total tumour populations under periodic or model-informed therapies are given in dashed grey or in solid blue, respectively. The drug sensitive ($\bar{A}(t)$) and drug tolerant ($\bar{B}(t)$) sub-populations under model informed therapy are shown in dot-dashed orange or dotted purple. The beginning of treatment on day 50 is denoted by a black star. The inset figure in Figure **C** shows the rapid decay of the M1 tumour population during therapy. Figures **B** and **D** show the ratio $\theta(t) = \bar{B}(t)/\bar{A}(t)$ during model-informed therapy in solid orange and the threshold ratio θ^* in dashed orange. Figure **E** illustrates the model-informed therapy where $\theta(t)$ is used to decide if therapy is given or not. The model parameters used in this simulation are given in Tables C and D.

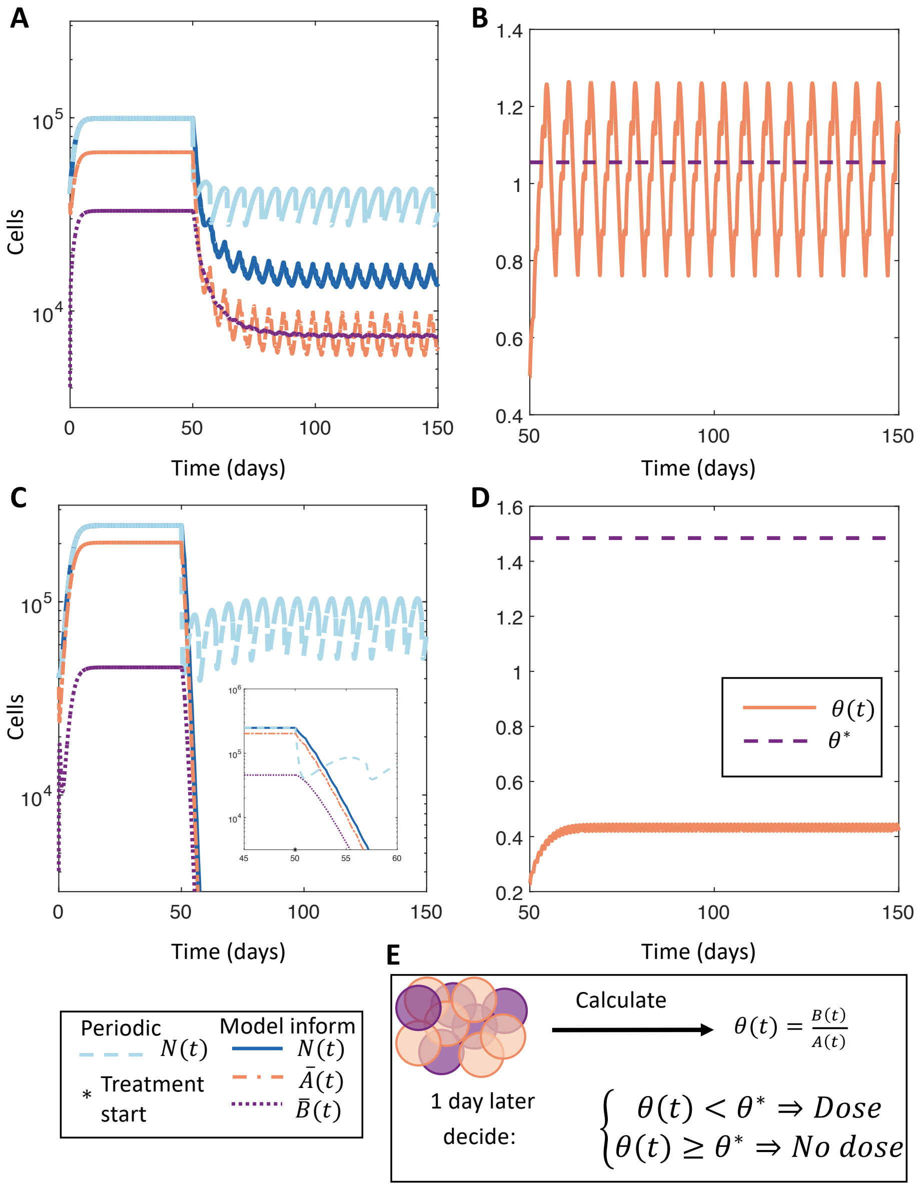

Fig I. Comparing model informed therapy and periodic dosing for bortezomib. Figures **A** and **C** compare model-informed therapeutic strategy with $T = 3$ against periodic treatment with bortezomib for the WT and M1 populations, respectively. The total tumour populations under periodic or model-informed therapies are given in dashed grey or in solid blue, respectively. The drug sensitive ($\bar{A}(t)$) and drug tolerant ($\bar{B}(t)$) sub-populations under model informed therapy are shown in dot-dashed orange or dotted purple. The beginning of treatment on day 50 is denoted by a black star. The inset figure in Figure **C** shows the rapid decay of the M1 tumour population during therapy. Figure **B** and **D** show the ratio $\theta(t) = \bar{B}(t)/\bar{A}(t)$ during model-informed therapy in solid orange and the threshold ratio θ^* in dashed orange. Figure **E** illustrates the model-informed therapy where $\theta(t)$ is used to decide if therapy is given or not. The model parameters used in this simulation are given in Tables C and D.

References

1. Proenca AM, Rang CU, Buetz C, Shi C, Chao L. Age structure landscapes emerge from the equilibrium between aging and rejuvenation in bacterial populations. Nature Communications. 2018;9(1):3722. doi:10.1038/s41467-018-06154-9.

2. Govers SK, Adam A, Blockeel H, Aertsen A. Rapid phenotypic individualization of bacterial sister cells. Scientific Reports. 2017;7(1):8473. doi:10.1038/s41598-017-08660-0.

3. Cassidy T, Craig M, Humphries AR. Equivalences between age structured models and state dependent distributed delay differential equations. Mathematical Biosciences and Engineering. 2019;16(5):5419–5450. doi:10.3934/mbe.2019270.

4. Perthame B. Transport Equations in Biology. Frontiers in Mathematics. Basel: Birkhäuser Basel; 2007. Available from: `http://www.amazon.com/exec/obidos/redirect?tag=citeulike07-20{&}path=ASIN/3764378417http://link.springer.com/10.1007/978-3-7643-7842-4`.

5. Rundnicki R, Mackey MC. Asymptotic Similarity and Malthusian Growth in Autonomous and Nonautonomous Populations. Journal of Mathematical Analysis and Applications. 1994;187(2):548–566. doi:10.1006/jmaa.1994.1374.

6. Billy F, Clairambaultt J, Fercoq O, Gaubertt S, Lepoutre T, Ouillon T, et al. Synchronisation and control of proliferation in cycling cell population models with age structure. Mathematics and Computers in Simulation. 2014;96:66–94. doi:10.1016/j.matcom.2012.03.005.

7. Cassidy T, Humphries AR. A mathematical model of viral oncology as an immuno-oncology instigator. Mathematical Medicine and Biology: A Journal of the IMA. 2019;To appear. doi:10.1093/imammb/dqz008.

8. Metz JAJ, Diekmann O, editors. The Dynamics of Physiologically Structured Populations. vol. 68 of Lecture Notes in Biomathematics. 3rd ed. Berlin, Heidelberg: Springer Berlin Heidelberg; 1986. Available from: `http://link.springer.com/10.1007/978-3-662-13159-6`.

9. Dingli D, Chalub FACC, Santos FC, Van Segbroeck S, Pacheco JM. Cancer phenotype as the outcome of an evolutionary game between normal and malignant cells. British Journal of Cancer. 2009;101(7):1130–1136. doi:10.1038/sj.bjc.6605288.

10. Ross-Gillespie A, Gardner A, Buckling A, West SA, Griffin AS. Density Dependence and CooperationL Theory and a Test with Bacteria. Evolution. 2009;63(9):2315–2325. doi:10.1111/j.1558-5646.2009.00723.x.

11. Korolev KS, Xavier JB, Gore J. Turning ecology and evolution against cancer. Nature Reviews Cancer. 2014;14(5):371–380. doi:10.1038/nrc3712.

12. Kimmel GJ, Gerlee P, Brown JS, Altrock PM. Neighborhood size-effects shape growing population dynamics in evolutionary public goods games. Communications Biology. 2019;2(1):53. doi:10.1038/s42003-019-0299-4.

13. Archetti M, Pienta KJ. Cooperation among cancer cells: applying game theory to cancer. Nature Reviews Cancer. 2019;19(2):110–117. doi:10.1038/s41568-018-0083-7.

14. Bacevic K, Noble R, Soffar A, Wael Ammar O, Boszonyik B, Prieto S, et al. Spatial competition constrains resistance to targeted cancer therapy. Nature Communications. 2017;8(1):1995. doi:10.1038/s41467-017-01516-1.

15. Greaves M, Maley CC. Clonal evolution in cancer. Nature. 2012;481(7381):306–313. doi:10.1038/nature10762.

16. Gatenby RA, Silva AS, Gillies RJ, Frieden BR. Adaptive Therapy. Cancer Research. 2009;69(11):4894–4903. doi:10.1158/0008-5472.CAN-08-3658.

17. Ben-Jacob E, S Coffey D, Levine H. Bacterial survival strategies suggest rethinking cancer cooperativity. Trends in Microbiology. 2012;20(9):403–410. doi:10.1016/j.tim.2012.06.001.

18. Gillies RJ, Brown JS, Anderson ARA, Gatenby RA. Eco-evolutionary causes and consequences of temporal changes in intratumoural blood flow. Nature Reviews Cancer. 2018;18(9):576–585. doi:10.1038/s41568-018-0030-7.

19. Altrock PM, Liu LL, Michor F. The mathematics of cancer: integrating quantitative models. Nature Reviews Cancer. 2015;15(12):730–745. doi:10.1038/nrc4029.

20. Jolly MK, Kulkarni P, Weninger K, Orban J, Levine H. Phenotypic Plasticity, Bet-Hedging, and Androgen Independence in Prostate Cancer: Role of Non-Genetic Heterogeneity. Frontiers in Oncology. 2018;8(MAR):1–12. doi:10.3389/fonc.2018.00050.

21. Nichol D, Robertson-Tessi M, Jeavons P, Anderson ARA. Stochasticity in the Genotype-Phenotype Map: Implications for the Robustness and Persistence of Bet-Hedging. Genetics. 2016;204(4):1523–1539. doi:10.1534/genetics.116.193474.

22. Zhang J, Cunningham JJ, Brown JS, Gatenby RA. Integrating evolutionary dynamics into treatment of metastatic castrate-resistant prostate cancer. Nature Communications. 2017;8(1):1816. doi:10.1038/s41467-017-01968-5.

23. Ramirez M, Rajaram S, Steininger RJ, Osipchuk D, Roth MA, Morinishi LS, et al. Diverse drug-resistance mechanisms can emerge from drug-tolerant cancer persister cells. Nature Communications. 2016;7(1):10690. doi:10.1038/ncomms10690.

24. Sharma SV, Lee DY, Li B, Quinlan MP, Takahashi F, Maheswaran S, et al. A Chromatin-Mediated Reversible Drug-Tolerant State in Cancer Cell Subpopulations. Cell. 2010;141(1):69–80. doi:10.1016/j.cell.2010.02.027.

25. Goldman A, Majumder B, Dhawan A, Ravi S, Goldman D, Kohandel M, et al. Temporally sequenced anticancer drugs overcome adaptive resistance by targeting a vulnerable chemotherapy-induced phenotypic transition. Nature Communications. 2015;6(1):6139. doi:10.1038/ncomms7139.

26. Cassidy T, Humphries AR, Craig M, Mackey MC. Characterizing Chemotherapy-Induced Neutropenia and Monocytopenia Through Mathematical Modelling. Bull Math Biol. 2020;82(8):104. doi:10.1007/s11538-020-00777-0.

27. Andersson DI, Hughes D. Antibiotic resistance and its cost: is it possible to reverse resistance? Nature Reviews Microbiology. 2010;8(4):260–271. doi:10.1038/nrmicro2319.

28. Inaba H. The Malthusian parameter and R_0 for heterogeneous populations in periodic environments. Mathematical Biosciences and Engineering. 2012;9(2):313–346. doi:10.3934/mbe.2012.9.313.

29. Diekmann O, Gyllenberg M, Metz JAJ. Finite dimensional state representation of linear and nonlinear delay systems. Journal of Dynamics and Differential Equations. 2018;30(4):1439–1467. doi:10.1007/s10884-017-9611-5.

30. Concepción-Acevedo J, Weiss HN, Chaudhry WN, Levin BR. Malthusian Parameters as Estimators of the Fitness of Microbes: A Cautionary Tale about the Low Side of High Throughput. PLOS ONE. 2015;10(6):e0126915. doi:10.1371/journal.pone.0126915.

31. Diekmann O, Heesterbeek JAP, Metz JAJ. On the definition and the computation of the basic reproduction ratio R 0 in models for infectious diseases in heterogeneous populations. Journal of Mathematical Biology. 1990;28(4):365–382. doi:10.1007/BF00178324.

32. Inaba H, Nishiura H. The state-reproduction number for a multistate class age structured epidemic system and its application to the asymptomatic transmission model. Mathematical Biosciences. 2008;216(1):77–89. doi:10.1016/j.mbs.2008.08.005.

33. Inaba H. On the definition and the computation of the type-reproduction number T for structured populations in heterogeneous environments. Journal of Mathematical Biology. 2013;66(4-5):1065–1097. doi:10.1007/s00285-012-0522-0.

34. Lewis K. Persister cells, dormancy and infectious disease. Nature Reviews Microbiology. 2007;5(1):48–56. doi:10.1038/nrmicro1557.

35. Keren I, Kaldalu N, Spoering A, Wang Y, Lewis K. Persister cells and tolerance to antimicrobials. FEMS Microbiology Letters. 2004;230(1):13–18. doi:10.1016/S0378-1097(03)00856-5.

36. Dingli D, Offord C, Myers R, Peng KW, Carr TW, Josic K, et al. Dynamics of multiple myeloma tumor therapy with a recombinant measles virus. Cancer Gene Therapy. 2009;16(12):873–882. doi:10.1038/cgt.2009.40.

37. MATLAB. R2017a. Natick, Massachusetts: The MathWorks Inc.; 2017.

38. Nichol D, Robertson-Tessi M, Anderson ARA, Jeavons P. Model genotype–phenotype mappings and the algorithmic structure of evolution. Journal of The Royal Society Interface. 2019;16(160):20190332. doi:10.1098/rsif.2019.0332.

39. Craig M, Kaveh K, Woosley A, Brown AS, Goldman D, Eton E, et al. Cooperative adaptation to therapy (CAT) confers resistance in heterogeneous non-small cell lung cancer. PLOS Computational Biology. 2019;15(8):e1007278. doi:10.1371/journal.pcbi.1007278.

40. Chen S, Xue Y, Wu X, Le C, Bhutkar A, Bell EL, et al. Global microRNA depletion suppresses tumor angiogenesis. Genes Dev. 2014;28(10):1054–1067. doi:10.1101/gad.239681.114.

41. Yang HW, Chung M, Kudo T, Meyer T. Competing memories of mitogen and p53 signalling control cell-cycle entry. Nature. 2017;549(7672):404–408. doi:10.1038/nature23880.

42. Bacaër N, Ait Dads EH. Genealogy with seasonality, the basic reproduction number, and the influenza pandemic. Journal of Mathematical Biology. 2011;62(5):741–762. doi:10.1007/s00285-010-0354-8.

43. Baker SD, Zhao M, Lee CKK, Verweij J, Zabelina Y, Brahmer JR, et al. Comparative Pharmacokinetics of Weekly and Every-Three-Weeks Docetaxel. Clinical Cancer Research. 2004;10(6):1976–1983. doi:10.1158/1078-0432.CCR-0842-03.

44. Bissery MC. Preclinical pharmacology of docetaxel. European Journal of Cancer. 1995;31(1004):S1–S6. doi:10.1016/0959-8049(95)00357-O.

45. Clarke SJ, Rivory LP. Clinical Pharmacokinetics of Docetaxel. Clinical Pharmacokinetics. 1999;36(2):99–114. doi:10.2165/00003088-199936020-00002.

46. Wind S, Schnell D, Ebner T, Freiwald M, Stopfer P. Clinical Pharmacokinetics and Pharmacodynamics of Afatinib. Clinical Pharmacokinetics. 2017;56(3):235–250. doi:10.1007/s40262-016-0440-1.

47. Agency EM. Giotrif: summary of product characteristics; 2018. Available from: `https://www.ema.europa.eu/en/documents/product-information/giotrif-epar-product-information{_}en.pdf`.

48. Tan CRC, Abdul-Majeed S, Cael B, Barta SK. Clinical Pharmacokinetics and Pharmacodynamics of Bortezomib. Clinical Pharmacokinetics. 2019;58(2):157–168. doi:10.1007/s40262-018-0679-9.
