# Supplementary material for: The role of memory in non-genetic inheritance and its impact on cancer treatment resistance
Source: PLoS Comput Biol. 2021 Aug 30;17(8):e1009348. doi: 10.1371/journal.pcbi.1009348 (PMC8432806; doi:10.1371/journal.pcbi.1009348)
Supplement: Attachment — Submitted filename: REVIEW_Theroleofmemory.pdf [file pcbi.1009348.s002.pdf]

Review: The role of memory in non-genetic inheritance and its impact on cancer treatment resistance

Summary

The authors propose a novel treatment model that incorporates two different populations, one which undergoes phenotypic switching and one which does not. Their results show differing types of resulting resistance and how different optimal treatment regimens would be required. This paper highlights clearly how understanding the presence of phenotype switching and applying the appropriate treatment for such populations is fundamental to optimising treatment. This was a really nicely written paper with an interesting discussion of phenotypic switching and an elegant underlying model. The implications and utility of the model are well discussed by the authors. The paper is only held back by some minor issues in communication, particularly as the paper reads as if written in a different order and rearranged/reduced without enough consideration for clarity. It is worth noting that their supplementary materials are extremely clear but some of this information still needs moving into the main text and some concepts need to be introduced before certain variables are reported or plotted. Furthermore, the code used for the modelling/production of graphs in this paper must be published in line with PlosCB requirements.

Main general points:

In general the symbols in equations are not defined early enough in the text, they are only defined in the materials and methods. An earlier statement of the definitions of all variables is required and would improve readability. Many of the specific points below relate to this general issue.

We suggest that a schematic diagram/cartoon of the model early in the paper with all parameters and key equations defined would significantly improve the impact and readability of this paper.

Additionally, figure captions and the text often do not address the figures in enough detail. Adding the points we have suggested will allow much easier reading and immediately address initial questions a reader will have.

The code used for the modelling in this paper must be published in line with PlosCB requirements.

Specific points

In the definition of equation (1) all of the terms are negative, we believe this is due to the production of new daughter cells at time t with age *a=0,* although this point is mentioned in the supplementary some of this explanation should ideally be moved to the main text to explain to the reader at first sight how/why this general form differs from classical ODE/PDE models.

The fact that $\bar{A}$ and $\bar{B}$ are the total number of each phenotype should be stated at first appearance in line **105.**

Between lines **127-128** the line numbers are missing, however the key point in this section is that in this paragraph σ_A **and** σ_B are not defined.

In **Fig 1** the authors should adjust the plot area so that it is square, this adjustment will emphasise the result that the plot is symmetric. The authors should also reproduce the scale bar so that the accompanying labels are not stretched. The addition of isoclines would also improve this figure.

Line 167 should read “less important *than* the probability”

There is a discontinuity in the second derivative in **Fig 2B**. The authors should check that this discontinuity is real and if so, discuss this discontinuity. The axes and legend labels should be larger for readability.

Fig 3 is partly cropped on both sides in the print format of the article. Labels should be increased in size. The blue line, N(t) is actually plotted as a *proportion of the carrying capacity*, this should be stated. We also believe that the length of treatment is discussed later on but the caption should include brief details on the treatment length derivation/ and a reference to supplementary.

Table 1 - Theta is not defined here, only much later. This table could be extended with symbolic definitions above and the second half of the table defining how selecting parameters was done. This problem could also be solved by including parameter definitions in a proposed cartoon of the model in the suggested figure.

Line 234 - Although the authors defined Cooperative Adaptation to Therapy in a previous paper, a refresh on the definition here would allow easier reading and improve the flow of this paragraph.

Line 307 / Equation above - $C_{1/2}Vol$ is defined in the supplementary/later but not at first sight, same for k_{elim}.

Fig 4 - Axis labelled as A+B cells but becomes <<1 (axes say 10^-5 / 10^-10) which is not physical for a pure sum. Address this and importantly define the quantities plotted on the axes in the plot caption.

Additionally, there is a repeated change in population size at 50 days, possibly due to treatment application or delayed response time? Something about this time should clearly be stated in the text and caption, we were unable to identify anywhere this was mentioned. This figure might benefit from having the treatment protocol overlayed on these plots. "Given in S3 table" should read given in Table S3. Table S3 also seems to show results derived from the plot in Fig 4 and not the parameters used to make the model which seem to appear in S1 and S2. We would appreciate clarification from the authors.

Time (days) repeated under figure part D in print version

In the **discussion**, we recommend the following paper be cited, Robert Vander Velde et al. *Resistance to targeted therapies as a multifactorial, gradual adaptation to inhibitor specific selective pressures. (https://www.nature.com/articles/s41467-020-16212-w)* This paper contains a significant investigation into the emergence of resistance in NSCLC, although under different drug treatments of Alectinib, Lorlatinib and Crizotinib, the paper presents resistance as a multifactorial, gradual process which is a result of relevance in the context of the phenotype switching model the authors propose.

Line 391 - should read "*or* drug sensitive"

We believe that the explanation in **lines 402-431** about the data from citation [7] is overly wordy and should focus simply on the relevant details to the model, rather than the methodology. This section needs to make extra clear that this was not work that was done by the authors for this paper. Changing the initial sentence to read "We used the previously published data from _ et al." would make this more explicit. We recommend that some of this experimental detail is instead mentioned in the text or in supplementary, when discussing the model/figures/fitting, as opposed to including such detail here which may give the false impression the experiment was carried out for this paper and does less to contextualise results in the text.

Line 433 "initial conditions corresponding to populations in exponential growth". Make sure that the exact conditions you're referring to are explicitly stated in the supplementary and are referenced in the main text.

Equations: C(t) $C_{1/2}$ not defined, R/r

Equation above **Line 438 :** closing bracket on limit is missing

k_{elim}, C(t) and $C_{1/2}$ are eventually defined here but their definitions must appear much earlier in the text in order to be understood in the results

Line 488 "curves *correspond*"
