# Supplementary material for: The role of memory in non-genetic inheritance and its impact on cancer treatment resistance
Source: PLoS Comput Biol. 2021 Aug 30;17(8):e1009348. doi: 10.1371/journal.pcbi.1009348 (PMC8432806; doi:10.1371/journal.pcbi.1009348)
Supplement: Attachment — Submitted filename: PlosCBReviewerComments.pdf [file pcbi.1009348.s003.pdf]

We are very grateful to all reviewers for their thoughtful comments, enthusiasm, and detailed reading of the manuscript. In what follows, we have **bolded** the reviewer comments and written our response in plain text. We have included quotes directly from the updated manuscript in *italicized text* and changes from the initial submission are bolded in the revised manuscript.

Reviewer 1

The authors have developed a stochastic model that tracks phenotypic switching of cells between their drug-resistant and drug-sensitive states, and demonstrate how this switching can influence the timescale of emergence and maintenance of drug tolerance in a phenotypically heterogeneous population. Based on fitting their simulation profiles to in vitro data, they also identify therapeutic strategies that can lead to sustained decay of tumor size without exhibiting long-term resistance. The study is well-done overall and caters to the emerging theme of non-genetic heterogeneity in enabling drug resistance/tolerance. I have the following request for authors to clarify some of their model assumptions and interpretations:

We are very grateful to the reviewer for their support and detailed reading of the manuscript. In particular, we appreciate their comments regarding the motivation of some of our model assumptions and formulation, which we feel have improved the manuscript.

1. If I understood correctly, the authors allow for phenotypic switching only at cell division, right? They should include a schematic as Fig 1 to explain more clearly their modeling framework. Also, is one of the inherent assumptions that both drug-tolerant and drug-sensitive cells have equal switching rate (or propensity in a continuum framework) to one another? Also, the authors should explain the existence of terms $R_A(A(t), B(t))$ and $R_B(A(t)$ in equation (3).

The reviewer is correct that we only allow for phenotypic switching at division and assume that phenotypes are fixed at birth. It would be possible to allow for cells to change phenotype throughout their lifetime but this would considerably complicate the model. We do not assume that drug tolerant and drug sensitive cells are equally likely to switch phenotype upon reproduction. Rather, we model the probability that a mother cell with phenotype i and age a produces two daughter cells with phenotype i as $\beta_{ii}(a)$. We updated the manuscript to read on page 5:

The probability of a cell with phenotype A and age a producing two daughter cells with the same phenotype is given by

$$\beta_{AA}(a) = P^*_{AA} + (P^{max}_{AA} - P^*_{AA}) \exp(-\sigma_A a),$$

while the probability of a cell of phenotype B with age a producing two cells of phenotype B is

$$\beta_{BB}(a) = P^*_{BB} + (P^{max}_{BB} - P^*_{BB}) \exp(-\sigma_B a).$$

In both cases, P^{max}_{ii} and P^*_{ii} are the maximal and minimal probabilities that a cell with phenotype i produces a cell with the same phenotype, respectively, but are specific to each phenotype.

We have included a cartoon schematic of the model as Figure 1 in the revised manuscript to add further clarity.

2. In their modeling framework, is the role of "age" similar to that of lineage tracing/barcoding a cell, i.e. counting for how many simulation time steps has an individual cell been around? Is age the only parameter that influences the switching rate/propensity? What is the connection of age with permanent vs. temporary resistance in the framework?

In our model framework, cellular age represents the chronological age of the cell, or the time since birth. While it would be possible to included other epigenetic factors to bias phenotype inheritance, this would

severely complicate the model. We have updated the manuscript to explicitly define "age" and explain it's use as a cipher for other epigenetic factors:

The structure variable a in (2) corresponds to chronological cellular age which we use as a cipher for a number of epigenetic factors. It would be possible to include these epigenetic factors by including a non-constant ageing velocity as in other structured models of physiological processes [3, 4]. However, it is difficult to determine how these epigenetic factors accumulate throughout a cell's lifespan and during treatment. Consequently, including a non-constant ageing velocity would severely complicate the formulation, parametrization, and analysis of (2) and would limit the utility of our simple model. Consequently, we only consider chronological age here.

In this framework, there is no explicit link between cellular age and treatment resistance. However, as we use cellular age to influence the likelihood of phenotype inheritance, there is an indirect link between the age of a reproducing cell and the likelihood that the offspring will retain the parent cell's sensitivity to treatment. This indirect link between age and resistance is illustrated in our results regarding permanent and transient resistance to treatment in Figure 4. However, this relationship is a result of the model rather than an assumption.

3. How are age and "memory" related? Do the authors define "memory" of a cell as its ability to maintain a phenotype upon cell division? Usually, the concept of "cell memory" is invoked upon to indicate hysteresis in a given system (Jolly and Celia-Terrassa, J Clin Med 2019).

We are grateful to the reviewer for making this important point. Hysteresis is an important aspect of any system that exhibits switching between states [2]. Most of our existing modelling work has considered phenotypic memory as the result of an explicit bi-stable chemical switch [14, 15]. Here, we aimed to relax the assumption of an explicit switch by replacing it with the functions β_{ii}. These functions act to bias the likelihood of daughter cells inheriting the phenotype of the mother cell rather than acting as an all-or-nothing event, with the model parameters P_{ii}^{max}, P_{ii}^{*} and σ_i acting as an analogue of the specific switching mechanism. This biased phenotypic inheritance represents the interaction between cellular age and memory of the mother's phenotype. Given how changes in inheritance strategies impact the model's response to therapy via the presence of bi-stability (as shown in Fig. 4), investigating the impact of hysteresis, via different functional forms of β, on model predictions would be an interesting extension of this work. However, it would come at the cost of analytical tractability.

4. The authors should clarify how many different parameters were fitted to the experimental data, and how many time points and conditions are needed to identify those number of parameters, without overfitting?

We are grateful to the reviewer for raising this point. As described in the *Methods,* there are five parameters to be fit to experimental data. Indeed, we show in the SI that these parameters need not be identifiable as, for a given switching strategy, there is a set of growth parameters that allow the model to fit experimental data. However, we also show that the switching strategy becomes apparent following therapy. Accordingly, when fitting experimental data, we simultaneously consider treated and untreated data. We have added the following text to the *Methods* section to emphasize this point:

The parameters remaining to be fit control either population growth (r_A, r_B, and d_A), or the probability of retaining the drug-tolerant phenotype (P_{BB}^{*} and P_{BB}^{max}). Thus, there are 5 parameters to be fit to experimental data.

We show in the Supporting Information that these parameters may not be identifiable for untreated data. In particular, for a given pair (P_{BB}^{*}, P_{BB}^{max}), it is possible to fit the parameters r_A, r_B and d_A can be chosen to fit experimental data equally well in the absence of treatment. However, the role of the parameters (P_{BB}^{*}, P_{BB}^{max}) becomes evident once therapy is administered and the previously indistinguishable curves become distinct.

5. The authors claim using ref 44 and 45 that with age, the rate of switching increases. However, both ref 44 and 45 do not seem to show this directly. Also, both of them are in

bacterial systems, not cancer cells. Can the authors provide stronger evidence for this key assumption in their model? Also, the authors provide the results for "stay" strategy using values of P_{BB} and $P_{BB_{max}}$ close to one another as well as close to 1; what happens for values say 0.45, 0.5?

The reviewer raises an excellent point regarding using experimental results from bacterial cells as evidence of phenotypic switching in cancer. There is evidence of phenotype switching in cancer drug resistance (see for example [7, 10, 17, 21, 23]). In particular, [22] demonstrated the heritability of rare gene expression patterns that are associated with treatment resistance in melanoma [21] which corresponds to phenotypic memory in our modelling formulation. However, the precise mechanism underlying phenotype switching has not yet been elucidated, despite the work by Yang et al. [28] that demonstrated that inheritance of p53 and mitogen-induced cyclin D1 acts to bias human mammary epithelial cells towards a proliferative or quiescent phenotype. As cyclin D1 is an unstable protein, increased duration of mother cell's life span may induce a bias toward quiescent daughter cells. In fact, [13] showed that a mild increase in the duration of the cell cycle induces a bias towards quiescent daughter cells in an immortalized non-transformed human epithelial cell line, MCF10A. Experimental work has shown that cells who enter the quiescent stage are likely to have mother cells who had a longer intermitotic time, which corresponds to an older mother cell in our modelling framework [1, 24, 26]. Here, it is likely the decreased degradation of cyclin D1 that biases the proliferation-quiescent switch, rather the increased intermitotic time. However, we use the increased intermitotic time, and thus older mother cells, to act as a cipher for these signalling factors. We have emphasized this in the text on page 5 which now reads:

We assumed that the probability of changing phenotypes depended on the age of the parent cell (i.e., older mother cells are more likely to have daughter cells that switch phenotypes [1, 26], where we recall that we are using chronological age as a surrogate for the degradation of cellular signalling pathways [8, 13, 18, 28])

Finally, our work indicates that the mechanisms of phenotypic switching (via different memory strategies) manifest themselves as observable population level behaviours in response to environmental change (i.e. treatment). In this perspective, our model can be viewed as a hypothesis that makes testable predictions and, thus, could prove useful for identifying model cancers/cell lines which exhibit switching and memory in the future. This would help identify cell lines that exhibit phenotypic memory and possibly contribute to the elucidation of the mechanisms underlying phenotypic memory in cancer cell systems.

If the value of P_{BB}^{max} is much smaller than one, then daughter cells are unlikely to have the same phenotype as mother cells so the strategy is more "switch" than stay. However, for $P_{BB}^{*} = 0.45, 0.5$, the switching strategy is similar to the "switch" strategy illustrated in the text, and so are the results.

6. The authors should comment on similarities and differences of their model formulation and key results with other recent efforts - Sahoo et al. bioRxiv 2021, Gunnarsson et al. J Theor Bio 2020).

We have added the following sentence to the discussion:

In this sense, our work addresses similar questions to [9, 20] although via a different and complementary axis, particularly in our analytical results and the development of the model-informed treatment.

and the following phrase to the introduction:

Recent modelling efforts used gene-regulatory networks or branching-type formulations to investigate the role of phenotypic switching on treatment resistance[9, 20], while other authors have used Markov processes to illuminate the role of stochastic phenotypic switching in treatment resistance [10, 17, 25].

Reviewer 2

The authors propose a mathematical model to investigate the role of phenotypic plasticity in treatment resistance, and to investigate treatment strategies to avoid establishment of drug-resistant phenotypes. It is shown that a model-informed therapy could drive tumor to extinction while preventing the risk of development of resistance. The paper is very well written and the mathematics is elegant and impressive. The model is simple but effective to illustrate important biological mechanisms. I only list very minor details below, that the authors may or may not take into account.

We are very grateful to the reviewer for their enthusiasm and detailed reading of the manuscript.

- **Supplementary vs Supplemental throughout the text.**

We have made this change and now use Supporting Information throughout.

- **Line 105: could be worth to explain the overline notation here already; another minor comment is that it could be worth to mention how reproduction is intended, i.e., that at rate R_A cells die and two daughter cells are born.**

We agree with the reviewer so we have introduced the bar notation at the beginning of the results in Equation (1) and have added the following text to explain how reproduction is included in our model:

As dividing cells necessarily have age $a > 0$, cellular reproduction results in the the disappearance of the mother cell, which accounts for the negative sign on the RHS of (2). As cellular division results in the production of 2 daughter cells with age $a = 0$, these reproducing cells re-enter the model through the boundary condition for $A(t, 0)$ and $B(t, 0)$. Accordingly, we model cellular reproduction through the non-local boundary conditions given in (3). These boundary conditions account for the production of daughter cells with age $a = 0$ from all dividing mother cells with age $a > 0$ through the integration in a.

We have also included a cartoon representation of the model in Figure 1 to further clarify this point.

- **Line 114: maybe mention that n is a parameter that describes the type of response**

We are grateful to the reviewer for spotting this omission and added the following sentence to the text

We use a Hill function formulation of $f_n(\bar{A}(t), \bar{B}(t))$ with Hill coefficient n that modulates the type of Allee effect

- **Eq (3): may be clearer to use brackets to isolate the argument of the integral.**

We agree with the reviewer and have made this change.

- **P. 4, before the definition of $beta_{AA}$, I would find it clearer to use "is assumed to be" rather than "given by", to make it clear that this is a model assumption. Also, please mention that sigma are positive parameters (incidentally I was curious why P^* is not denoted by P^{min}, but not necessary to change)**

We agree with the reviewer and have changed the sentence to use "is assumed to be" and have mentioned that the σ_i are strictly positive parameters. We use P^* to denote the long-term probability of inheriting the phenotype and have mentioned this on page 5.

- **I find Figure 1 little informative, considering that the shown behavior is quite intuitive. Maybe some extra explanation to stress what you want to show?**

In the revised manuscript, we now show the Malthusian parameter as a function of λ_A and λ_B for three different switching strategies, including the *stay* and *switch* strategies considered later. By considering the isoclines, we see how different switching strategies influence how increases in the fitness of a sub-population impact the fitness of the mixed population. We have updated the figure caption to highlight this relationship and have added the following text to the manuscript:

We first note that the population level fitness is an increasing function of the fitness of the subpopulations, λ_A and λ_B, as we would expect. Further inspection of Fig. (2) illustrates how the different switching strategies influence the role of fitness increases in each constituent sub-population on fitness of the entire population. In particular, we note that in the stay strategy, fitness increases in the drug tolerant population are more impactful on the entire population than the drug sensitive population, while the opposite is in true for the stay strategy.

- **Fig 2 caption: isn't this for increasing values of the SENSITIVE cell death rate? In the legend, I find 11/30 and 19/30 less intuitive to interpret as the decimal notation, I'm not sure if there was a reason for this but it is unclear; it may be worth to use the same scale in vertical axis in panels A-B and panels C-D**

We are very grateful to the reviewer for catching the typo in the caption. We have corrected this mistake in the revised manuscript. We now list the decimal values for each parameter curve and have plotted all the figures on the same vertical axis.

- **Line 184-186: how is the σ_B fixed?**

We fix $\sigma_B = \sigma_A = 10^{-2}$ throughout. We discuss this choice (on page 1 of the SI) and perform sensitivity analysis on this choice of parameter on page 22 of the Supporting Information.

- Line 187: ... of the POPULATION carrying capacity K (in order to also define K).

We have made this change.

- **Line 194: I am not familiar with the term "objective response rate"**

The objective response rate is the proportion of patients whose tumour burden decreased during therapy and is often used in evaluating cancer treatments [11, 12, 19]. We added the following text to the manuscript on page 9:

objective response rate, or proportion of patients with tumour reduction following therapy, ...

- **Line 207: "the drug-tolerant population became dominant": this sentence is unclear to me, as the left panel shows that the proportion of drug tolerant cells is only 20%, hence it doesn't seem to me to be dominant. Maybe clarify what you mean?**

We are grateful to the reviewer for pointing out this inaccurate sentence. We meant that the drug-tolerant population was dominant during therapy and have updated the sentence to read:

For both the switch and stay strategies, the tumour population eventually developed resistance as the drug-tolerant phenotype became dominant during treatment

- **Line 208: in the switch population in Fig 3A, sensitive cells seem to remain above 40% (not just above** 20%)

The reviewer is correct and we have made this change.

- **Figure 3 (and results): it is unclear at this stage what parameters are used for the drug-sensitive population (β_{AA}). May be useful to include a table?**

We are grateful to the reviewer for catching this. We have added Table S1 with the parameters used for this figure. The figure caption now indicates that the parameters used in this figure are given in Table S1.

- **Eq (5): is n the same parameter defining the Allee effect? If so, it may be useful to briefly recall it.**

We have made this change and the text now reads

To accomplish this, the ratio of drug-tolerant to drug-sensitive cells, $\bar{B}/\bar{A}$, must not exceed the threshold

ratio ϑ_ε^* which is given by

$$\vartheta_\varepsilon^* = \left[\frac{\varepsilon r_A - r_B}{r_A(1-\varepsilon)}\right]^{1/n}, \tag{0.1}$$

where we recall that the parameter n determines the strength of the Allee effect.

- Line 434 "Generic model of chemotherapy": I think this section would better be located before the "Numerical simulation of phenotypic switching model", as it defines the variable C(t) and related quantities, which are otherwise not defined in the ODE system presented at p. 15. I would also specify that this describes the standard PERIODIC treatment mentioned in the results.

We agree with the reviewer and have moved the generic model of chemotherapy to the Results section. We have also emphasized that we are using this model when simulating treatment.

We are very grateful to the reviewer for identifying these typos. We have corrected them (and others).

- Line 167: that -> than.

- Table 1, seventh entry: Ratio B/A (rather than A/B)? last entry: "such that $\lambda_B(\theta) < 0$", add: for $\theta < \theta^*$

- Line 127: leads to THE following.

- Line 270: a approximately -> an approximately.

- Line 384: it's -> its.

- Equation after (9): bracket is missing from interval.

- Lines 465 and 467: "r_A" is listed twice in the two lists – typo?.

In the Supplementary Material:

- P. 1 line 39: "after 1 day will HAVE"

- P. 2 line 76, "nutrient" (typo)

- P. 3 line 77 "carrying"

- Four lines after (S8): relative fitness OF these cells

- Equation after (S10): a closing bracket is missing in the interval

- P. 5, three lines after definition of N_{AA} : not sure if "either" is in the correct position in the sentence

- Equation after (S14): R_I should be R_A ?

- P. 7 second line of the equation for N_{AA} in (S15): there is an argument "ts" (typo)

- P. 25 last equation: comma rather than full stop

- P. 28, line 4: $f_N \to f_n$

- P. 19 line 371: extra closing bracket

In the Supplementary Material:

After (S5), "As expected" sounds strange as I thought this was the assumption leading to the choice of the beta functions

We agree with the reviewer and have erased this phrase from the sentence.

- P. 9 line 166: an stable -> a stable (typo). You should also probably mention that the eigenproblem is studied for the linearization of (S2), or alternatively for constant growth rates rA and rB ?

We have corrected this typo and added the following sentence emphasizing that we are working with the linearized version of S2:

This stable age distribution is equivalent to finding the first eigenelements of (S2) where we are considering the linearised version of (S2). We note that the linearised version of (S2) corresponds to purely Malthusian growth corresponding to constant R_A and R_B and effectively unlimited resources.

- P. 24, line after (S32): are you here assuming unconstrained growth $R_i = r_i$?

The reviewer is correct and we have added the following sentence to emphasize this point:

As in our calculation of the stable age distribution, we once again consider the linearised version of S32 corresponding to Malthusian growth.

Reviewer 3

Summary
The authors propose a novel treatment model that incorporates two different populations, one which undergoes phenotypic switching and one which does not. Their results show differing types of resulting resistance and how different optimal treatment regimens would be required. This paper highlights clearly how understanding the presence of phenotype switching and applying the appropriate treatment for such populations is fundamental to optimising treatment. This was a really nicely written paper with an interesting discussion of phenotypic switching and an elegant underlying model. The implications and utility of the model are well discussed by the authors. The paper is only held back by some minor issues in communication, particularly as the paper reads as if written in a different order and rearranged/reduced without enough consideration for clarity. It is worth noting that their supplementary materials are extremely clear but some of this information still needs moving into the main text and some concepts need to be introduced before certain variables are reported or plotted. Furthermore, the code used for the modelling/production of graphs in this paper must be published in line with PlosCB requirements.

We are very grateful to the reviewer for their enthusiasm about the manuscript, in particular, their comments and detailed reading identified areas for improvement in presentation and communication.

Main general points:

In general the symbols in equations are not defined early enough in the text, they are only defined in the materials and methods. An earlier statement of the definitions of all variables is required and would improve readability. Many of the specific points below relate to this general issue.

We suggest that a schematic diagram/cartoon of the model early in the paper with all parameters and key equations defined would significantly improve the impact and readability of this paper.

We re-wrote the *Phenotypic switching model* section of the results in the updated manuscript. In particular, we state the model variables and parameters before they appear in the equations on page 4. We included the generic chemotherapy model in the *Phenotypic switching model* section to introduce the parameters *Dose* and $C_{1/2}$ before their appearance later in the manuscript. We have also included a cartoon representation of the model in Fig. 1.

Additionally, figure captions and the text often do not address the figures in enough detail.

We have re-written the figure captions in the updated manuscript.

Adding the points we have suggested will allow much easier reading and immediately address

initial questions a reader will have.

The code used for the modelling in this paper must be published in line with PlosCB requirements.

Portions of the code were written while TC was employed at the Los Alamos National Laboratory (LANL). Accordingly, LANL must approve the release of source code which requires approval from a number of internal and external organizations including the U.S. Department of Energy and the U.S. National Nuclear Security Administration. We have begun the export control process which is expected to take several months.

The current manuscript was submitted March 1st, 2021 while the PLOS CB requirements for code sharing only apply to manuscripts submitted after March 31st, 2021. However, in the interest of reproducibility, we will release the source code upon approval from LANL and will link the source code from personal websites. The released code will be available at *https://github.com/lanl/phenotype-switching*. In the interim, we have added a section to the Supporting Information discussing the implementation of the model in Matlab.

Specific points

In the definition of equation (1) all of the terms are negative, we believe this is due to the production of new daughter cells at time t with age a=0, although this point is mentioned in the supplementary some of this explanation should ideally be moved to the main text to explain to the reader at first sight how/why this general form differs from classical ODE/PDE models.

We agree that this may be confusing and have added the following explanation of the negative terms to the main text:

As dividing cells necessarily have age $a > 0$, cellular reproduction results in the the removal of the mother cell, which accounts for the negative sign on the RHS of (2). As cellular division results in the production of 2 daughter cells with age $a = 0$, these reproducing cells re-enter the model through the boundary condition for $A(t, 0)$ and $B(t, 0)$. Accordingly, we model cellular reproduction through the non-local boundary conditions given in (3). These boundary conditions account for the production of daughter cells with age $a = 0$ from all dividing mother cells with age $a > 0$ through the integration over the age variable a.

We emphasized this point in the caption of Fig. 1 to explain why the RHS of (2) is negative. We have also added the following discussion regarding differences between ODE and PDE models:

While the distinction between disappearance of mother cells and the appearance of daughter cells is natural in age-structured populations such as (2) [3, 16], it results in a strictly negative RHS of (2). Since general ODE models consider a homogeneous population of cells without accounting for cellular age, there is no distinction made between the division (and subsequent removal) of a mother cell and the appearance of daughter cells. Accordingly, ODE models only include the net population gain due to reproduction, i.e. each mother cell producing two daughter cells, which typically results in a non-negative term and differs from the distinction made between division of a mother cell and production of daughter cells in the age structured model (2). This fundamental difference results from the inclusion of biological information, such as cellular age, and the resulting population heterogeneity in structured PDE models which is generally not possible in the ODE framework.

The fact that $\bar{A}$ and $\bar{B}$ are the total number of each phenotype should be stated at first appearance in line 105.

The definition of $\bar{A}$ and $\bar{B}$ is now in equation (1) and reads:

The object of clinical interest at time t is unlikely to be the density of cells with a given age, but rather total number of cells of each phenotype, given by

$$\bar{A}(t) = \int_0^\infty A(t, a)\mathrm{d}a \quad \text{and} \quad \bar{B}(t) = \int_0^\infty B(t, a)\mathrm{d}a. \tag{0.2}$$

In what follows, the total number of cells is denoted by $N(t) = \bar{A}(t) + \bar{B}(t)$.

Between lines 127-128 the line numbers are missing, however the key point in this section is that in this paragraph σ_A and σ_B are not defined.

We have added the following definition of σ_i to the main text on page 5:

The parameter σ_i represents the decay rate of intracellular signalling factors and modulates how ageing impacts the probability of daughter cells retaining the mother cells phenotype. We enforce $\sigma_i > 0$

In Fig 1 the authors should adjust the plot area so that it is square, this adjustment will emphasise the result that the plot is symmetric. The authors should also reproduce the scale bar so that the accompanying labels are not stretched. The addition of isoclines would also improve this figure.

In the revised manuscript, we now show the Malthusian parameter as a function of λ_A and λ_B for three different switching strategies, including the *stay* and *switch* strategies considered later. Following the reviewers suggestion to include the isoclines, we see how different switching strategies influence how increases in the fitness of a sub-population impact the fitness of the mixed population. Finally, each subplot is square. We have updated the figure caption to highlight this relationship and have added the following text to the manuscript:

We first note that the population level fitness is an increasing function of the fitness of the subpopulations, λ_A and λ_B, as we would expect. Further inspection of Fig. (2) illustrates how the different switching strategies influence the role of fitness increases in each constituent sub-population on fitness of the entire population. In particular, we note that in the stay strategy, fitness increases in the drug tolerant population are more impactful on the entire population than the drug sensitive population, while the opposite is in true for the stay strategy.

There is a discontinuity in the second derivative in Fig 2B. The authors should check that this discontinuity is real and if so, discuss this discontinuity. The axes and legend labels should be larger for readability.

We have re-generated the figures so that the axis labels and legends are legible. We have also re-written the caption to better contextualise the figures.

Upon further inspection, the discontinuity in the second derivative of Fig 2B was an artifiact of the numerical root finding technique used to calculate the stable age distribution. In essence, the stable age distribution is calculated from the eigenvector v that solves the eigenvalue problem $M(\lambda_P)v = 1v$ as we show in Section *Stable Age Distribution and Population Proportion* on page 17 of the SI (where the eigenvalue is 1 since λ_P is the Malthusian parameter). We initially calculated the stable age distribution by solving the equivalent system of equations $(M - 1I)v = 0$. The discontinuity in Fig 2B resulted from the root finding algorithm calculating $v = 0$ (to numerical precision) as a solution of the system of equations. However, this solution is not an eigenvector and so does not define the stable age distribution. In the revised version, we specifically solve the Eigenvalue-Eigenvector formulation derived in the SI using established numerical techniques rather than the mathematically equivalent root finding problem. We regret this error.

Fig 3 is partly cropped on both sides in the print format of the article. Labels should be increased in size. The blue line, $N(t)$ is actually plotted as a proportion of the carrying capacity, this should be stated. We also believe that the length of treatment is discussed later on but the caption should include brief details on the treatment length derivation/ and a reference to supplementary.

In the updated manuscript, we re-generated the figures so that the axis labels are legible. We have also included information regarding the duration and scheduling of treatment in the caption. We corrected the figure legend in the updated manuscript.

Table 1 - θ is not defined here, only much later. This table could be extended with symbolic

definitions above and the second half of the table defining how selecting parameters was done. This problem could also be solved by including parameter definitions in a proposed cartoon of the model in the suggested figure.

We have moved the table later in the text so that all parameters are introduced before being listed in the table. We have also included the definition of θ and it's use in determining model-informed therapy in Fig. 5.

Line 234 - Although the authors defined Cooperative Adaptation to Therapy in a previous paper, a refresh on the definition here would allow easier reading and improve the flow of this paragraph.

We agree with the reviewer and have added the following sentence to briefly recall the definition of CAT to the main text:

During CAT, cancer cells behave co-operatively to induce drug tolerance in neighbouring cells and thus induce treatment resistance.

Fig 4 - Axis labelled as A+B cells but becomes « 1 (axes say $10^{-5}/10^{-10}$) which is not physical for a pure sum. Address this and importantly define the quantities plotted on the axes in the plot caption.

We are grateful to the reviewer for raising the issue with the non-physical sum observed in Figure 5C. The sustained tumour decay is due to the sustained drug pressure induced on the population that results in sustained tumour decay, eventually to undetectable levels. The model informed therapy is designed to induce $R_0^* < 1$ where R_0^* is the spectral radius of the next generator operator during treatment, so we expect to see exponential decay during therapy. This exponential decay will eventually result in tumour extinction, or the observed non-physical sums, if we are also able to avoid the establishment of a drug tolerant population. This is possible in both examples shown in the main text, which account for the small tumour sizes observed. In our initial submission, we included the long time simulations to illustrate this point, but these simulations included the non-physical tumour sizes mentioned by the reviewer.

In our revisions, we now include the dynamics of the ratio $\theta(t)$. The behaviour of this ratio with $\theta(t) < \theta^*$ is crucial in inhibiting the establishment of the drug tolerant population in Panel B and D of Fig 5. When combined with the dose size taken to ensure that $R_0^* < 1$, the dynamics of $\theta(t) < \theta^*$ indicate why we see sustained tumour decay. To emphasize this point, we have included the following text in the revised manuscript:

*In fact, the stable oscillations at (or below) the threshold ratio θ^* in Panels **B** and **D** of Fig. 5 combined with the exponential decay of $N(t)$ shown in Panels **A** and **C** illustrate the efficacy of the model informed therapy to preserve a sufficiently large population of sensitive cells while driving tumour extinction. This exponential decay is precisely what we expected from model-informed therapy where we consistently have a large enough population of sensitive cells, denoted by $\theta(t) < \theta^*$, as $R_0^* < 1$ during therapy (see the Supporting Information for details.)*

We defined the quantities plotted in the updated legend and figure caption and hope that the updated figure is more clear.

Additionally, there is a repeated change in population size at 50 days, possibly due to treatment application or delayed response time? Something about this time should clearly be stated in the text and caption, we were unable to identify anywhere this was mentioned. This figure might benefit from having the treatment protocol overlayed on these plots. "Given in S3 table" should read given in Table S3. Table S3 also seems to show results derived from the plot in Fig 4 and not the parameters used to make the model which seem to appear in S1 and S2. We would appreciate clarification from the authors.

We are grateful to the reviewer for catching this omission and mistake. The reviewers are correct that

the parameters used for these figures were given in S1 and S2 and that treatment was applied at day 50. Following the addition of two new tables corresponding to the generic model parameters and the best fit parameters for the Dingli et al. data in the SI, we have updated the references to the correct tables in the SI. We also updated the figure caption to illustrate that treatment begins on day 50 and give a cartoon representation of the model informed treatment schedule in the figure.

In the discussion, we recommend the following paper be cited, Robert Vander Velde et al. Resistance to targeted therapies as a multifactorial, gradual adaptation to inhibitor specific selective pressures. (https://www.nature.com/articles/s41467-020-16212-w) This paper contains a significant investigation into the emergence of resistance in NSCLC, although under different drug treatments of Alectinib, Lorlatinib and Crizotinib, the paper presents resistance as a multifactorial, gradual process which is a result of relevance in the context of the phenotype switching model the authors propose.

We are grateful to the reviewer for directing us to this very interesting paper and added the following text to the Discussion:

In particular, resistance to targeted therapies in NSCLC has been shown to result from a series of gradual epigenetic and genetic adaptations to treatment induced selection pressures [27].

and

The results of Vander Velde et al. [27] suggest implementing a continuous phenotype landscape in our model as well as extending our analysis to study combination therapies, strategies for drug combination, and the continued evolution of treatment resistance.

We believe that the explanation in lines 402-431 about the data from citation [7] is overly wordy and should focus simply on the relevant details to the model, rather than the methodology. This section needs to make extra clear that this was not work that was done by the authors for this paper. Changing the initial sentence to read "We used the previously published data from Craig et al." would make this more explicit. We recommend that some of this experimental detail is instead mentioned in the text or in supplementary, when discussing the model/figures/fitting, as opposed to including such detail here which may give the false impression the experiment was carried out for this paper and does less to contextualise results in the text.

We agree with the reviewer. The text now reads:

We used the previously published in vitro growth assay data from Craig et al. [6] to parametrize our mathematical model. Briefly, in their work, the parental (WT) cell line was derived from KRas-G12D, p53-/-, Dicer1f/- *genotype lung tumours and mutants (M1 and M2) were obtained through transfection to* Dicer1+/+ and Dicer1-/- using CRISPR-Cas9 [5]. *Cells were plated as tumour spheroids on NanoCulture plates and population growth without and with drug was assessed via flow cytometry on days 1, 3, 5, and 7 [6]*

with the remaining detail given in the SI.

Line 433 "initial conditions corresponding to populations in exponential growth". Make sure that the exact conditions you're referring to are explicitly stated in the supplementary and are referenced in the main text.

We are grateful to the reviewer for raising this point and catching this omission. We have added a specific section on the initial conditions of the ODE model to the SI and now give explicit expressions for the initial conditions of the four variables in the ODE formulation, as well as how they relate to the initial age distribution of cells in the PDE formulation.

1) Equation above Line 438 : closing bracket on limit is missing kelim, C(t) and C1/2 are eventually defined here but their definitions must appear much earlier in the text in order to be understood in the results. 2) Equations: C(t) C1/2 not defined, R/r. 3) Line 307 /

Equation above - C1/2Vol is defined in the supplementary/later but not at first sight, same for kelim.

We corrected the missing bracket and added the generic model of chemotherapy to the first section of the results so that these parameters are introduced earlier in the text.

We are very grateful to the reviewer for identifying the following typos. We have corrected them (and others) in the revised text.

Time (days) repeated under figure part D in print version

Line 391 - should read "or drug sensitive"

Line 488 "curves correspond

Line 167 should read "less important than the probability"

References

1 M. Arora, J. Moser, H. Phadke, A. A. Basha, and S. L. Spencer, *Endogenous Replication Stress in Mother Cells Leads to Quiescence of Daughter Cells*, Cell Rep., 19 (2017), pp. 1351–1364.

2 L. Cardelli and A. Csikász-Nagy, *The Cell Cycle Switch Computes Approximate Majority*, Sci. Rep., 2 (2012), p. 656.

3 T. Cassidy, M. Craig, and A. R. Humphries, *Equivalences between age structured models and state dependent distributed delay differential equations*, Math. Biosci. Eng., 16 (2019), pp. 5419–5450.

4 T. Cassidy, A. R. Humphries, M. Craig, and M. C. Mackey, *Characterizing Chemotherapy-Induced Neutropenia and Monocytopenia Through Mathematical Modelling*, Bull. Math. Biol., 82 (2020), p. 104.

5 S. Chen, Y. Xue, X. Wu, C. Le, A. Bhutkar, E. L. Bell, F. Zhang, R. Langer, and P. A. Sharp, *Global microRNA depletion suppresses tumor angiogenesis*, Genes Dev., 28 (2014), pp. 1054–1067.

6 M. Craig, K. Kaveh, A. Woosley, A. S. Brown, D. Goldman, E. Eton, R. M. Mehta, A. Dhawan, K. Arai, M. M. Rahman, S. Chen, M. A. Nowak, and A. Goldman, *Cooperative adaptation to therapy (CAT) confers resistance in heterogeneous non-small cell lung cancer*, PLOS Comput. Biol., 15 (2019), p. e1007278.

7 A. Goldman, B. Majumder, A. Dhawan, S. Ravi, D. Goldman, M. Kohandel, P. K. Majumder, and S. Sengupta, *Temporally sequenced anticancer drugs overcome adaptive resistance by targeting a vulnerable chemotherapy-induced phenotypic transition*, Nat. Commun., 6 (2015), p. 6139.

8 S. K. Govers, A. Adam, H. Blockeel, and A. Aertsen, *Rapid phenotypic individualization of bacterial sister cells*, Sci. Rep., 7 (2017), p. 8473.

9 E. B. Gunnarsson, S. De, K. Leder, and J. Foo, *Understanding the role of phenotypic switching in cancer drug resistance*, J. Theor. Biol., 490 (2020), p. 110162.

10 P. B. Gupta, C. M. Fillmore, G. Jiang, S. D. Shapira, K. Tao, C. Kuperwasser, and E. S. Lander, *Stochastic State Transitions Give Rise to Phenotypic Equilibrium in Populations of Cancer Cells*, Cell, 146 (2011), pp. 633–644.

11 A. Hoos, C. M. Britten, C. Huber, and J. O'Donnell-Tormey, *A methodological framework to enhance the clinical success of cancer immunotherapy*, Nat. Biotechnol., 29 (2011), pp. 867–870.

12 I. Mellman, G. Coukos, and G. Dranoff, *Cancer immunotherapy comes of age*, Nature, 480 (2011), pp. 480–489.

13 M. Min, Y. Rong, C. Tian, and S. L. Spencer, *Temporal integration of mitogen history in mother cells controls proliferation of daughter cells*, Science (80-.)., 368 (2020), pp. 1261–1265.

14 D. Nichol, M. Robertson-Tessi, A. R. A. Anderson, and P. Jeavons, *Model genotypeÔÇôphenotype mappings and the algorithmic structure of evolution*, J. R. Soc. Interface, 16 (2019), p. 20190332.

15 D. Nichol, M. Robertson-Tessi, P. Jeavons, and A. R. Anderson, *Stochasticity in the Genotype-Phenotype Map: Implications for the Robustness and Persistence of Bet-Hedging*, Genetics, 204 (2016), pp. 1523–1539.

16 B. Perthame, *Transport Equations in Biology*, Frontiers in Mathematics, Birkhäuser Basel, Basel, 2007.

17 A. O. Pisco, A. Brock, J. Zhou, A. Moor, M. Mojtahedi, D. Jackson, and S. Huang, *Non-Darwinian dynamics in therapy-induced cancer drug resistance*, Nat. Commun., 4 (2013), p. 2467.

18 A. M. Proenca, C. U. Rang, C. Buetz, C. Shi, and L. Chao, *Age structure landscapes emerge from the equilibrium between aging and rejuvenation in bacterial populations*, Nat. Commun., 9 (2018), p. 3722.

19 H. Rehman, A. W. Silk, M. P. Kane, and H. L. Kaufman, *Into the clinic: Talimogene laherparepvec (T-VEC), a first-in-class intratumoral oncolytic viral therapy*, J. Immunother. Cancer, 4 (2016), pp. 1–8.

20 S. Sahoo, A. Mishra, H. Kaur, K. Hari, S. Muralidharan, S. Mandal, and M. Kumar Jolly, *A mechanistic model captures the emergence and implications of non-genetic heterogeneity and reversible drug resistance in ER+ breast cancer cells Running title: EMT and therapy resistance in ER+ breast cancer cells*, bioRxiv, (2021), p. 2021.03.14.435359.

21 S. M. Shaffer, M. C. Dunagin, S. R. Torborg, E. A. Torre, B. Emert, C. Krepler, M. Beqiri, K. Sproesser, P. A. Brafford, M. Xiao, E. Eggan, I. N. Anastopoulos, C. A. Vargas-Garcia, A. Singh, K. L. Nathanson, M. Herlyn, and A. Raj, *Rare cell variability and drug-induced reprogramming as a mode of cancer drug resistance*, Nature, 546 (2017), pp. 431–435.

22 S. M. Shaffer, B. L. Emert, R. A. Reyes Hueros, C. Cote, G. Harmange, D. L. Schaff, A. E. Sizemore, R. Gupte, E. Torre, A. Singh, D. S. Bassett, and A. Raj, *Memory Sequencing Reveals Heritable Single-Cell Gene Expression Programs Associated with Distinct Cellular Behaviors*, Cell, 182 (2020), pp. 947–959.e17.

23 S. V. Sharma, D. Y. Lee, B. Li, M. P. Quinlan, F. Takahashi, S. Maheswaran, U. McDermott, N. Azizian, L. Zou, M. A. Fischbach, K. K. Wong, K. Brandstetter, B. Wittner, S. Ramaswamy, M. Classon, and J. Settleman, *A Chromatin-Mediated Reversible Drug-Tolerant State in Cancer Cell Subpopulations*, Cell, 141 (2010), pp. 69–80.

24 S. L. Spencer, S. D. Cappell, F.-C. Tsai, K. W. Overton, C. L. Wang, and T. Meyer, *The Proliferation-Quiescence Decision Is Controlled by a Bifurcation in CDK2 Activity at Mitotic Exit*, Cell, 155 (2013), pp. 369–383.

25 Y. Su, W. Wei, L. Robert, M. Xue, J. Tsoi, A. Garcia-Diaz, B. Homet Moreno, J. Kim, R. H. Ng, J. W. Lee, R. C. Koya, B. Comin-Anduix, T. G. Graeber, A. Ribas, and J. R. Heath, *Single-cell analysis resolves the cell state transition and signaling dynamics associated with melanoma drug-induced resistance*, Proc. Natl. Acad. Sci., 114 (2017), pp. 13679–13684.

26 Y. Uetake and G. Sluder, *Prolonged Prometaphase Blocks Daughter Cell Proliferation Despite Normal Completion of Mitosis*, Curr. Biol., 20 (2010), pp. 1666–1671.

27 R. Vander Velde, N. Yoon, V. Marusyk, A. Durmaz, A. Dhawan, D. Miroshnychenko, D. Lozano-Peral, B. Desai, O. Balynska, J. Poleszhuk, L. Kenian, M. Teng, M. Abazeed, O. Mian, A. C. Tan, E. Haura, J. Scott, and A. Marusyk, *Resistance to targeted therapies as a multifactorial, gradual adaptation to inhibitor specific selective pressures*, Nat. Commun., 11 (2020), p. 2393.

28 H. W. Yang, M. Chung, T. Kudo, and T. Meyer, *Competing memories of mitogen and p53 signalling control cell-cycle entry*, Nature, 549 (2017), pp. 404–408.
